# Organ‐on‐a‐Chip Technology: Advances in Modeling Inflammation, Neurovascularization and Nociception in Human Intervertebral Disc Degeneration

**DOI:** 10.1002/smmd.70051

**Published:** 2026-08-28

**Authors:** Izzat Zulkiflee, Nurul Fariha Za'aba, Abhay Pandit, Isma Liza Mohd Isa

**Affiliations:** ^1^ CÚRAM Research Ireland Centre for Medical Devices University of Galway Galway Ireland; ^2^ Department of Anatomy Faculty of Medicine Universiti Kebangsaan Malaysia Kuala Lumpur Malaysia; ^3^ Pharmacology and Therapeutics University of Galway Galway Ireland

**Keywords:** hydrogel, inflammation, intervertebral disc degeneration, microfluidic, nociception, organ‐on‐a‐chip

## Abstract

An organ‐on‐a‐chip platform has emerged as an advanced in vitro model that replicates organ‐level structures within physiologically relevant human microenvironments. In this review, we focus on intervertebral disc (IVD) degeneration, a leading cause of chronic low back pain, providing a comprehensive overview of the current understanding of IVD anatomy, physiology, and pathogenesis. We review conventional in vitro, ex vivo, and in vivo models, which have expanded our understanding; however, they inadequately reproduce the human phenotype, heterogeneous disc composition and anatomy, which comprises nucleus pulposus (NP) and annulus fibrosus (AF) with neovascularization and sensory innervation within the three‐dimensional (3D) degenerative microenvironment. We emphasize a disc‐on‐a‐chip platform to address these limitations, which integrates microfluidics and biomimetic ECM‐based hydrogels within a controlled microenvironment to miniaturize the disc. It offers a promising platform to simulate the pathological disc milieu by incorporating gradient control of key signaling molecules implicated in disc degeneration. These include a disintegrin and metalloproteinase with thrombospondin motifs (ADAMTS) and matrix metalloproteinase (MMP)‐mediated extracellular matrix (ECM) degradation; tumor necrosis factor alpha (TNF‐α) and interleukin‐1 beta (IL‐1β)‐driven inflammation via nuclear factor kappa‐light‐chain‐enhancer of activated B cells (NF‐κB); vascular endothelial growth factor (VEGF)‐promoted angiogenesis; and nerve growth factor (NGF)‐ and brain‐derived neurotrophic factor (BDNF)‐mediated sensory innervation and sensitization, thus contributing to nociception. Collectively, disc‐on‐a‐chip offers a next‐generation in vitro model for investigating mechanisms relating to ECM degradation, inflammation, innervation, vascularization, and nociception underlying IVD degeneration, enabling precision preclinical therapeutic evaluations.

## Highlights

- Clinical significance of IVD degeneration, a primary etiological factor of chronic low back pain, represents a major global musculoskeletal health burden. It involves cellular phenotype alteration, molecular and structural changes, affecting spine biomechanics.
- Organ‐on‐a‐chip technology replicates organ‐level structures within physiologically relevant human microenvironments, offering a next‐generation preclinical platform to model disease mechanisms underlying IVD degeneration and therapeutic testing over conventional models.
- The disc‐on‐a‐chip platform integrates microfluidics and biomimetic ECM‐based hydrogels to miniaturize the IVD within a controlled microenvironment while maintaining the human IVD phenotype.
- Disc‐on‐a‐chip enables gradient control of key signaling mediators implicated in disc degeneration, simulating the pathological disc milieu, including ECM degradation, inflammation, neovascularization, sensory innervation, and nociception.

## Graphical abstract

Organ‐on‐a‐chip to miniaturize the human intervertebral disc in modeling key degenerative features, including ECM degradation, inflammation, neovascularization, sensory innervation, and nociception toward precision translation for studying disease mechanisms and therapeutic testing.

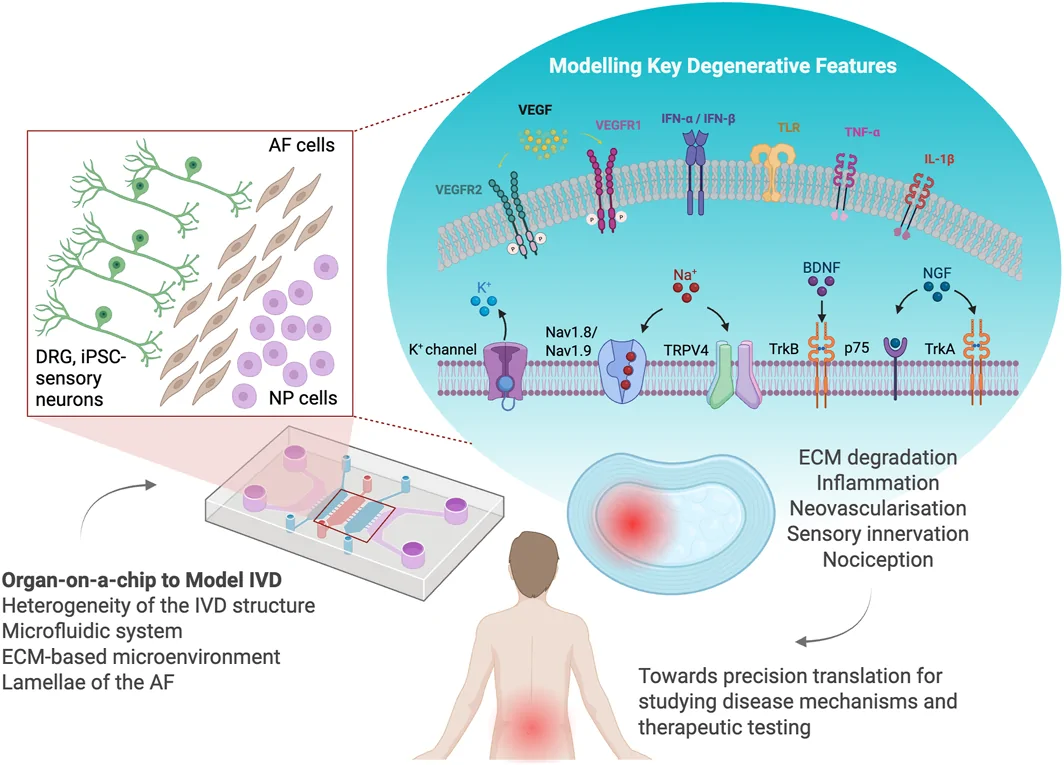

## 1 Introduction

Intervertebral disc (IVD) degeneration is one of the primary etiologies of chronic low back pain, which affects 619 million people globally and poses a challenging clinical issue [1]. The prevalence of IVD degeneration increases with age, with studies showing that up to 90% of individuals over 60 years of age exhibit some degree of disc degeneration [2, 3, 4]. Beyond aging, risk factors include genetics, obesity, and lifestyle factors, such as sedentary behavior. IVD degeneration not only limits physical function but also imposes substantial economic burdens on healthcare systems due to medical costs and lost workdays [5]. It is characterized by an altered cellular phenotype, causing gradual structural and biochemical alterations, resulting in extracellular matrix (ECM) degradation and inflammation. An increase in inflammation progresses with prolonged ECM degradation, which induces nociceptive nerve innervation into the aneural disc, contributing to pain [6]. The severity of IVD degeneration increases with inflammation, not only impairing disc function but also causing pain, stiffness, and mobility problems [7]. A feedback loop in which inflammation worsens degeneration and degeneration perpetuates inflammation. Despite this understanding, research is hindered by the lack of precise diagnostic tools for early detection and the complexity of targeting inflammatory pathways underlying IVD degeneration. To improve outcomes, a comprehensive understanding of the molecular mechanisms driving IVD degeneration is crucial to developing therapies that address both the symptoms and root causes of the condition.

Research on IVD degeneration has long relied on animal models and traditional in vitro systems; however, these approaches have significant limitations. Animal models often fail to accurately replicate the unique anatomical, biomechanical, and cellular phenotype of the human IVD. Moreover, species‐specific differences in disc size, composition, and injury response limit the development of therapies tailored to human physiology. Similarly, conventional in vitro models, such as two‐dimensional (2D) cell cultures, lack the complexity of the three‐dimensional (3D) microenvironment of native IVDs. While traditional in vitro and in vivo models have provided valuable insights into the pathophysiology of IVD degeneration, their inability to capture the multifaceted nature of inflammation and nociception pathophysiology in IVD degeneration has necessitated the development of novel techniques.

Novel approaches to the study of IVD degeneration have been made possible by recent developments in organ‐on‐a‐chip technology. The disc‐on‐a‐chip model, a micro‐engineered human IVD, provides a physiologically relevant platform in a controlled microenvironment for modeling the pathophysiology of inflammation, innervation, and nociception underlying IVD degeneration [8]. By integrating key features of organ‐on‐a‐chip technology, such as microfluidics and 3D ECM‐based hydrogels, and incorporating human‐derived cells in controlled microenvironments, disc‐on‐a‐chip can accurately recapitulate the pathophysiology of human IVD degeneration. It allows multicellular interactions by modulating biochemical cues, such as inflammatory and neurogenic mediators and biomechanical loading, to model inflammation, innervation and nociception, which are involved in mechanisms underlying IVD degeneration. Disc‐on‐a‐chip enables real‐time analysis of cellular responses under pathophysiological degenerative conditions and improves the predictive accuracy of therapeutic testing, thereby addressing the gap between laboratory studies and clinical applications. It represents a novel in vitro platform for investigating the mechanisms underlying painful intervertebral disc degeneration, enabling mechanistic disease modeling and precise therapeutic evaluation.

We review the current understanding of the anatomy and physiology of the IVD and the pathogenesis of disc degeneration, focusing on key mechanisms underlying painful IVD degeneration. We highlight existing models for in vitro, ex vivo and in vivo studies of IVD degeneration and the current state‐of‐the‐art of organ‐on‐a‐chip technology within the context of IVD degeneration. We emphasize advancing our understanding of inflammation‐mediated degeneration and nociceptive signaling in device development, including strategies to fabricate the disc‐on‐a‐chip and cell integrations. Unlike previous reviews that solely focused on biological mechanisms of IVD degeneration or on organ‐on‐a‐chip technology independently, this review comprehensively integrates both mechanisms underlying painful IVD degeneration and biomimetic disc‐on‐a‐chip engineering, offering a comprehensive framework for human‐relevant IVD disease modeling on ECM degradation, inflammation, neovascularization, innervation and nociception and translational therapeutic development. We conclude the review by discussing the impact of disc‐on‐a‐chip in precision disease modeling, mechanistic discovery, and translational applications, while outlining its challenges and future direction.

## 2 Intervertebral Disc: Anatomy and Physiology

**FIGURE 1:**
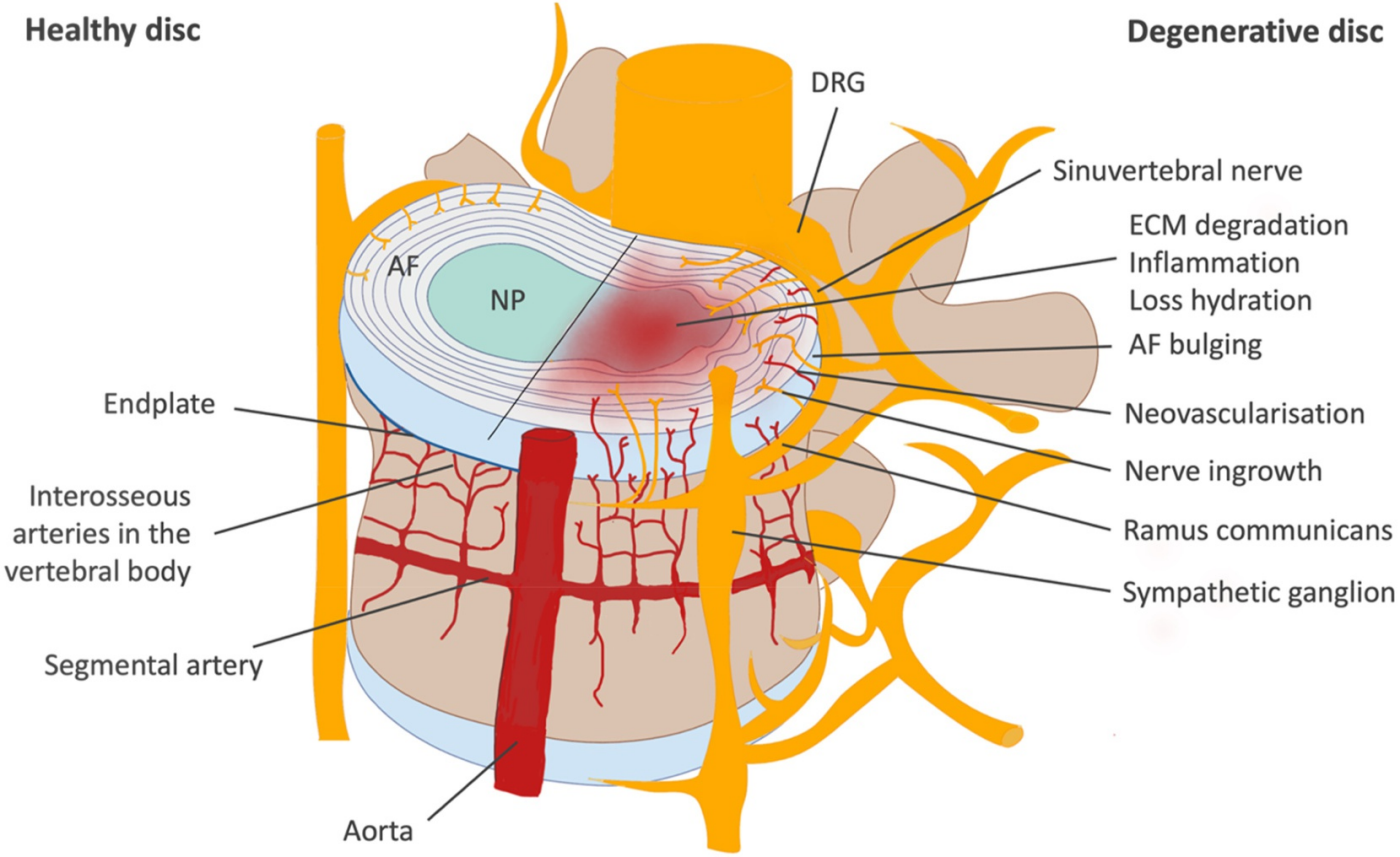
Structural and pathological changes associated with intervertebral disc degeneration. Schematic illustration comparing a healthy and degenerative intervertebral disc (IVD). The healthy IVD consists of the gelatinous NP surrounded by concentric AF lamellae and exhibits limited vascularization and innervation at the outer AF. During degeneration, ECM degradation, inflammation, tissue hydration and structural disruption promote neurovascular ingrowth into the disc, contributing to nociceptor sensitization and chronic discogenic pain. Created in https://BioRender.com.

The IVD is a cartilaginous joint between adjacent vertebrae that provides mechanical stability, acts as a shock absorber, and allows movement such as flexion, extension, side bending, and rotation at the motion segments [9, 10]. It consists of the gelatinous proteoglycan‐rich inner core nucleus pulposus (NP) and collagenous lamellae of annulus fibrosus (AF). Cartilaginous and nutrient‐regulating tissue of cartilage endplates (CEP) is sandwiched superiorly and inferiorly between the IVDs [11] (Figure 1). Embryologically, the IVD is derived from the notochord and sclerotome, the NP originates from the notochord, and the AF and CEP arise from the sclerotome [12].

### 2.1 Nucleus Pulposus

The NP mainly lies in the central and posterior parts of the disc. It is a gel‐like, highly hydrated structure with low cell density (∼3000 cells/mm^3^), mainly composed of small chondrocyte‐like nucleopulpocytes and larger vacuolated notochordal cells (NCs) [13]. The early response gene EGR1 plays a key role in regulating degeneration‐related genes, underscoring the impact of NP cell heterogeneity on disc degeneration [12]. The ECM of NP consists of 70%–90% water with its dry weight composed of approximately 35%–65% proteoglycans, 5%–20% fine type II collagen fibrils, and smaller amounts of non‐collagenous proteins and elastin [14]. Its high water content, maintained by proteoglycans, helps distribute hydraulic pressure across the ECM, thereby reducing stress on the disc [14, 15]. NCs in the NP support disc health by promoting matrix production, protecting cells from degradation, and regulating other cell types, particularly during development [16]. During maturation, NC in the NP is replaced by chondrocyte‐like cells. As degeneration progresses, CLCs lose their ability to maintain tissue homeostasis, leading to their depletion. This results in reduced glycosaminoglycans (GAG) and water content and altered type II collagen to type I collagen. These changes, beginning with the loss of NCs, are closely linked to early disc degeneration [17, 18].

### 2.2 Annulus Fibrosus

AF is a fibrocartilaginous structure that forms the disc's outer layer and is typically divided into inner and outer regions [15]. The outer AF is rich in collagen type I and contains elongated, fibroblast‐like cells, whereas the inner AF shows increasing levels of collagen type II and proteoglycans, along with more rounded chondrocyte‐like cells [19]. The interlamellar spaces contain proteoglycans, elastic fibers, and cells. The AF consists of 65%–70% water, while its dry weight mainly includes collagen (50%–70%), proteoglycans (20%), and elastin (2%) [14, 20]. The AF provides mechanical support by containing the NP, preventing radial bulging, and allowing controlled spinal movements [21]. Its collagen fibrils anchor the disc to the adjacent vertebral end plates, helping to secure the disc in place and maintain spinal stability [15]. With aging, the AF experiences collagen disorganization, resulting in decreased stiffness, increased microdamage, and reduced mechanical strength. These alterations weaken the AF's capacity to withstand stress and strain [22].

### 2.3 Cartilaginous End Plate

The CEP covers the superior and inferior ends of the IVD [23]. It is approximately 600 μm thick with no variation across the disc levels, but is thinner centrally [24]. It is a hyaline cartilage layer located between the NP and the vertebral endplate. It serves as both a protective barrier and a channel for nutrient diffusion from the surrounding blood vessels [24]. CEPs help nourish the intervertebral disc by acting as a semi‐permeable barrier, allowing nutrients such as glucose and oxygen to diffuse from vertebral blood vessels into the largely avascular disc, supporting cell viability and function [23, 25]. It distributes mechanical loads and prevents disc bulging into the vertebral body [23]. Composed primarily of proteoglycans, type II collagen, and water, the CEP also aids in load transmission and NP containment [26]. Blood vessels are present in CEP channels during early life but regress by age 10, leaving adult CEPs largely avascular unless damaged [11]. During IVD degeneration, the CEP becomes sclerotic, loses its vascular connections, and exhibits reduced permeability [24].

### 2.4 Vascularization and Innervation in Intervertebral Disc

A healthy IVD is largely avascular with limited arterial supply to the outermost region of the AF. The remaining disc tissues depend on diffusion for the transport of nutrients and oxygen and the removal of metabolic waste, mainly through the vertebral bone via the adjacent CEP and the peripheral AF. Pathological changes affecting the CEP, such as bony sclerosis, altered blood flow, or endplate calcification, may impair this transport pathway, leading to cellular degeneration and death within the NP, the region most susceptible due to its distance from the vascular supply [11, 27]. The outer one‐third of the AF is innervated with small nerve fibers, whereas NP remains aneural under physiological conditions. During IVD degeneration, neurovascular ingrowth extends deeper into the disc, contributing to nociceptive signaling, as discussed in the next section. The anatomical and physiological attributes outline the essential biological properties that must be replicated in disc‐on‐a‐chip platforms to attain physiologically relevant models of IVD degeneration.

## 3 Pathophysiology of Intervertebral Disc Degeneration

**FIGURE 2:**
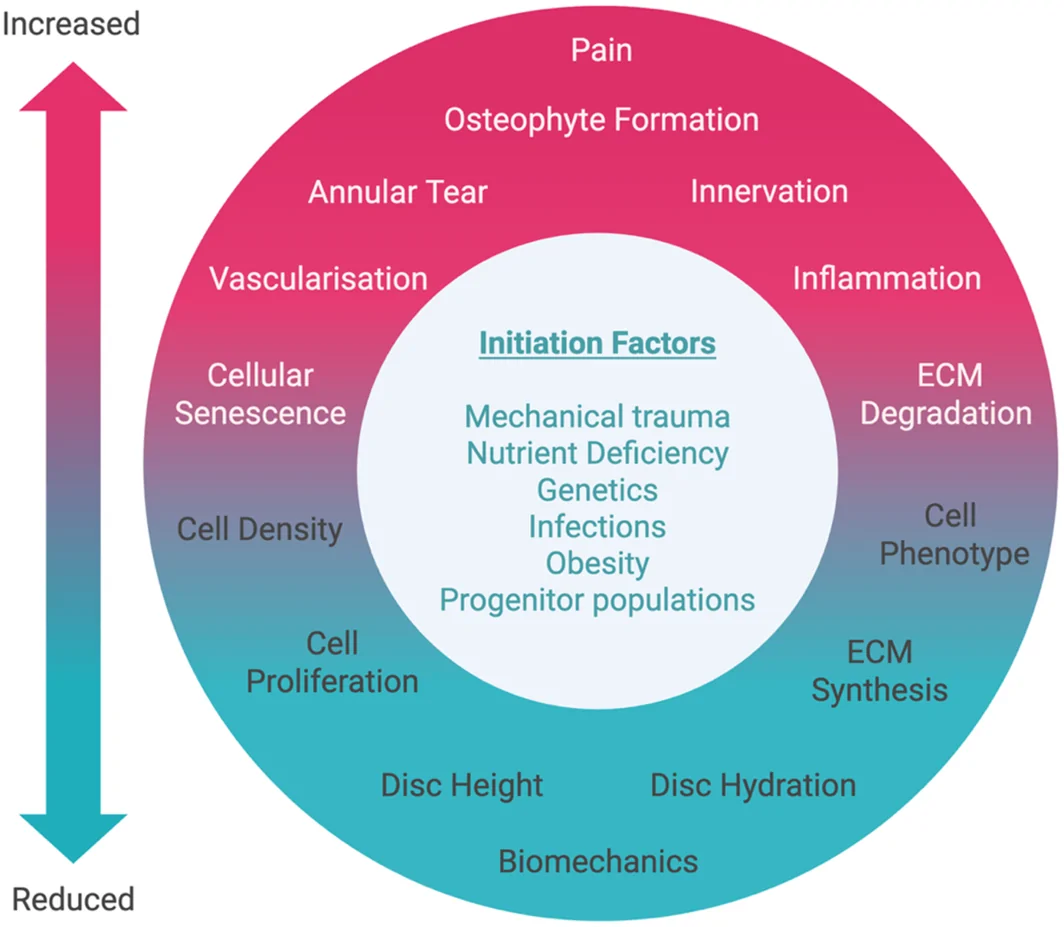
Progressive pathological changes during intervertebral disc degeneration. Schematic representation illustrating the multifactorial progression of IVD degeneration. Initiating factors trigger progressive alterations in disc cellularity, ECM homeostasis, hydration, biomechanics, inflammation, neurovascular ingrowth, and pain, highlighting the key pathological features relevant to the biomimetic disc‐on‐a‐chip model. Created in https://BioRender.com.

As the normal anatomy and physiology of the healthy IVD is established, the pathological changes that occur during degeneration are summarized in Figure 1. Intervertebral disc degeneration is initiated by multiple factors, including loss of progenitor cell populations, mechanical trauma, genetics, nutrient deficiency, infections, and obesity, which are at the core of the degenerative process, triggering a cascade of pathological changes (Figure 2). In the early stages of IVD degeneration, subtle biochemical and structural changes lay the foundation for progressive degeneration. The initial signs of IVD degeneration typically present as reduced levels of aggrecan and type II collagen in the NP, along with increased levels of pro‐inflammatory cytokines such as interleukin‐1 beta (IL‐1β) and tumor necrosis factor‐alpha (TNF‐α) [28]. These cytokines promote the activity of ECM‐degrading enzymes, specifically matrix metalloproteinases (MMPs) and A disintegrin and metalloproteinase with thrombospondin motifs (ADAMTS), thereby breaking down critical ECM structural components, such as collagen and proteoglycans [28].

As degeneration progresses, chronic inflammation becomes more pronounced, enhancing the degradation of the IVD matrix and structural instability. Chronic inflammation is promoted by the secretion of these pro‐inflammatory mediators, including TNF‐α, IL‐1β, and IL‐6, which are upregulated in degenerated IVD [29, 30, 31]. It contributes to the disruption of ECM homeostasis by increasing the activity of MMPs and aggrecanases, leading to further loss of proteoglycans and collagen, compromising the biomechanical integrity of the disc [32]. ECM breakdown products, such as fragmented proteoglycans, can function as damage‐associated molecular patterns (DAMPs), activating innate immune receptors on disc cells and prolonging inflammation [33, 34]. Cellular senescence and apoptosis become more prominent, exacerbating the inflammatory response and further reducing the number of viable disc cells capable of maintaining tissue homeostasis [35]. Neurogenic mediators also play a role in IVD degeneration‐associated pain at this stage, as pro‐inflammatory cytokines enhance the expression of nerve growth factor (NGF) and brain‐derived neurotrophic factor (BDNF), facilitating nociceptive nerve ingrowth into the aneural IVD [36]. These new growths of nociceptive nerve fibers, coupled with the increased local release of pain‐sensitizing mediators, such as substance P and calcitonin gene‐related peptide (CGRP), mediate nociception. Progressive ECM structural degradation combined with nerve sensitization results in mild and persistent pain [36, 37].

In severe degeneration, the neuroinflammatory microenvironment reaches a critical level, significantly impairing the mechanical function of the disc. Disc cells, along with infiltrating immune cells such as neutrophils, CD68+ macrophages, and T cells (CD4+ and CD8+), continue to secrete inflammatory cytokines and neurogenic factors, including NGF and BDNF [31]. The persistent upregulation of pro‐inflammatory cytokines, including TNF‐α, IL‐1β, and IL‐6, drives extensive ECM degradation, resulting in the AF tear and increasing susceptibility to disc herniation [29, 30, 31]. Pro‐inflammatory cytokines enhance NGF expression, facilitating aberrant nerve ingrowth, whereas pain‐sensitizing mediators, such as substance P and CGRP, further exacerbate nociception [36, 37]. Senescence and apoptosis exacerbate inflammation and degeneration, contributing to an irreversible decline in disc function [35]. Degradation of structural components, particularly collagen and proteoglycans, further reduces the biomechanical integrity of the disc, leading to spinal instability and increased stress on adjacent structures [32]. Degeneration‐induced disc height loss causes the ligamentum flavum to infold, the spinal canal to shorten, and the superior articular process of the facet joint to subluxate. This, in turn, alters lumbar lordosis, requiring compensatory postural adjustments, such as increased pelvic tilt and thoracic hyperlordosis, leading to increased muscular workload and fatigue‐related pain [38]. Patients frequently experience exacerbated pain while standing and find alleviation while resting, suggestive of mechanical discogenic pain [39]. The interplay between inflammation and nerve sensitization perpetuates a cycle of pain and degeneration.

## 4 Current Experimental Models of IVD Degeneration and Their Limitations

To study IVD degeneration, models that closely replicate the complex biochemical, mechanical, and inflammatory processes involved are required. Table 1 presents a comparative evaluation of various models used in IVD research. Various methods have been employed to simulate and study different aspects of IVD degeneration. These approaches aim to improve the understanding of the underlying mechanisms of degeneration and facilitate preclinical evaluation of novel treatments designed to alleviate pain, restore function, and improve biomechanics [40]. We summarize the current in vitro, ex vivo, and in vivo models in Table 2.

**TABLE 1:** Comparative evaluation of experimental models to study intervertebral disc degeneration.

Evaluation criteria	Conventional in vitro	Ex vivo organ culture	In vivo	Disc‐on‐a‐chip
Human relevance	++	+++	++	++++
Native 3D architecture	+	++++	++++	++++
Mechanical loading	+	+++	++++	+++
Oxygen/nutrient/pH gradients	+	++	++++	++++
ECM mimicry	++	++++	++++	++++
Immune component	+	+	++++	++
Vascular component	−	−	++++	++
Neuronal component	−	−	++++	++
Functional nociceptive assessment	−	−	++++	++
Experimental control	++++	++++	+	++++
Throughput	++++	+	+	+++
Standardization	++++	++	+	++
Cost‐effectiveness	++++	+++	+	+++
Major limitation	Oversimplified microenvironment	Limited tissue viability	Species differences	Fabrication complexity
References	[40, 41, 42, 43, 44, 45, 46, 47, 48, 49, 50, 51, 52, 53]	[54, 55, 56, 57, 58]	[41, 54, 55, 56, 57, 58, 59, 60, 61, 62]	[8, 42, 63, 64, 65, 66, 67, 68, 69, 70, 71, 72, 73, 74, 75, 76, 77]

*Note:* ++++ = excellent; +++ = high; ++ = moderate; + = limited; − = absent or not represented.

**TABLE 2:** Recent models of inflammation and sensory innervation have been developed based on their criteria for application.

Models	Cell sources	Biomaterials	Experimental approach	Key findings	Refs
In vitro					
Microfluidic	NP, AF, immune cells	Collagen I, GelMA	IL‐1β induction	Models induce disc cell degeneration, matrix breakdown, and senescence‐like phenotypes	[78]
				Elevation of pro‐inflammatory markers (IL‐6, IL‐8, TNF‐α) observed upon cytokine or immune‐cell stimulation. Provide quantifiable readouts of cell viability, ECM integrity, and inflammatory signaling	
Microfluidic	Human AF cells, macrophage‐like THP‐1 cells	Collagen I hydrogel	IL‐1β induction	Gradient IL‐1β and macrophage‐derived cytokines induced inflammatory and catabolic responses, increased IL‐6, IL‐8, MMP‐1, and MMP‐3 expression, promoted myofibroblast‐like morphology, angiogenesis‐associated signaling, and degeneration‐related kinetic changes in AF cells	[70]
2D cell culture	NP, AF	Standard cell culture	TNF‐α induction	TNF‐α induced pro‐inflammatory cytokine expression (IL‐6, IL‐8, MCP‐1). Increased markers of senescence and ECM degradation observed	[79]
2D cell culture	Rat NP cells	Maltol (MA)	IL‐1β induction	MA suppressed IL‐1β‐induced ECM degradation and inflammation by downregulating MMP‐3, MMP‐9, ADAMTS‐4/5, iNOS, COX‐2, IL‐1β, and IL‐18 while restoring aggrecan and collagen II expression through inhibition of PI3K/AKT/NF‐κB and NLRP3 inflammasome‐mediated pyroptosis	[80]
3D cell culture	Bovine NP cells	Catalase‐loaded polymer capsules functionalized with tannic acid (TA)	IL‐1β induction	IL‐1β‐induced inflammatory NP model showed increased oxidative stress and upregulation of MMP‐3 and ADAMTS‐5. Antioxidant polymer capsules reduced intracellular ROS, attenuated MMP‐3 and ADAMTS‐5 expression, and preserved NP cell viability	[81]
3D cell culture	Bovine NP cells	Hyaluronic acid (HA) Hydrogel with PEG	IL‐1β induction	HA hydrogels suppressed inflammatory receptors IL‐1R1 and MyD88, downregulated neurotrophins NGF and BDNF, maintained NP cell viability, and potentially exerted anti‐inflammatory effects through CD44‐mediated signaling	[82]
Ex vivo					
NP explant degeneration model	Bovine coccygeal NP explants	Notochordal cell‐derived matrix (NCM), chondroitin sulfate (CS)	Chondroitinase ABC (ChABC)‐induced degeneration with NCM or chondroitin sulfate treatment	chABC induced progressive sGAG depletion, reduced FCD and equilibrium stress, increased creep and matrix disorganization. NCM promoted cell proliferation and improved matrix organization but failed to restore biomechanical function due to sGAG leakage	[83]
Organ culture	Human MSC	Fibrin hydrogel	MSC‐seeded fibrin hydrogel cultured in healthy (high glucose) and degenerative (low glucose) human IVD explants	MSC response depended on disc degenerative state and carrier. MSCs in fibrin enhanced anabolic gene expression (COL2, ACAN) in degenerative discs. Physiological discs promoted greater MSC discogenic differentiation	[55]
Organ culture	Native human IVD cells	None	Long‐term human whole‐organ IVD culture with retained cartilage endplates (high or low glucose, high or low serum)	Retaining cartilage endplates preserved long‐term cell viability, limited swelling, maintained ECM integrity, and enabled long‐term human disc culture for regenerative studies	[84]
Organ culture	Native bovine NP and AF cells	Thermoreversible hyaluronan hydrogel	Thermoreversible hyaluronan hydrogel implantation under physiological loading	Developed AF and endplate cavity approaches for NP repair testing under physiological loading. Endplate approach improved sealing and disc height preservation, while AF approach caused greater height loss and tissue deformation	[56]
Organ culture	Native disc ECM	None	TNF‐α induction and TNF‐α inhibitor	TNF‐α stimulation led to regional variation in inflammation markers. Detected localized matrix breakdown, cell death, and cytokine expression. Preserved physiological features of disc degeneration over time	[79]
Bovine, organ‐level system	NP, AF within ECM	None	TNF‐α induction with anti‐inflammatory drug treatments	TNF‐α induced catabolic responses, including increased gene expression of MMPs, ADAMTS, and inflammatory cytokines. Disc tissue showed reduced proteoglycan content, indicative of matrix degradation. Tested anti‐inflammatory drugs (e.g., IL‐1Ra, etanercept) successfully mitigated inflammatory responses in a dose‐dependent manner	[58]
Bovine, bioreactor system	NP, AF within ECM	None	CPP‐miRNA treatment in a bovine bioreactor culture system	Treatment with CPP–miRNA complexes reduced expression of inflammatory cytokines (e.g., IL‐6, IL‐8). Upregulation of regenerative markers and ECM components (e.g., aggrecan, collagen II) observed. Demonstrated cell penetration and gene modulation within intact disc tissue	[85]
In vivo					
Sham injection IVDD animal models	Native IVD cells	None	Needle puncture injury using different needle diameters with PBS sham control	Disc degeneration severity depended on needle diameter‐to‐disc height ratio. Needle:disc ratios > 40% consistently induced degeneration, whereas < 25% caused minimal mechanical and structural changes	[86]
Rabbit	Native rabbit IVD cells	HA hydrogel with PEG	Surgically induced IVDD followed by HA hydrogel with PEG treatment	HA hydrogel maintained disc height, increased T1ρ/T2 MRI hydration signals, promoted NP cellularity and organized AF lamellae, while suppressing IL‐6 expression and nociceptive nerve in growth in degenerative discs	[87]
Rat	Native NP and AF cells	HA/COLII hydrogel	Surgically induced IVDD treated with HA/COLII hydrogel	HA/COLII hydrogel alleviated mechanical allodynia, inhibited GAP43‐positive sensory hyperinnervation, and restored disc hydration shown by increased MRI T1ρ values following surgically induced disc injury	[88]
Rat	Native NP and AF cells	HA Hydrogel	Surgically induced IVDD treated with HA hydrogel	Reduced thermal hyperalgesia and mechanical allodynia, suppressed sensory hyperinnervation (GAP43, CGRP, TRPV1, Trk‐A), decreased IL‐1β/IL‐6 signaling, and improved ECM remodeling after disc injury	[89]
Mice	NP cells	Maltol (MA)	Maltol (MA) treatment in an IVDD mouse model	MA reduced disc degeneration, proteoglycan loss, MMP‐9, p‐PI3K, p‐p65, and NLRP3 expression while preserving collagen II and disc structural integrity in vivo	[80]
Rat	NP, AF	None, native disc tissue	Needle puncture‐induced IVDD model	Upregulation of inflammatory cytokines (IL‐1β, TNF‐α) and sensory‐related genes is observed in disc tissue. Structural changes in the disc include loss of disc height, annular fissures, and endplate disruption	[61]
Rat	NP, AF	None, native disc tissue	Needle puncture‐induced IVDD model	Induced histological disc degeneration, including decreased proteoglycan content, reduced disc height, and matrix disorganization	[62]
				Rats developed behavioral hypersensitivity and pain‐like responses, indicating successful modeling of discogenic pain. Elevated levels of inflammatory mediators and nerve ingrowth‐related markers observed in disc tissue	

### 4.1 In Vitro Models

A recent study showed that adipose‐derived stem cells differentiated into chondrocyte‐like cells when co‐cultured with chondrocytes and stimulated by native nucleus NP cells, highlighting a promising approach for IVD regeneration [90]. These models provide significant insights into the reactions of NP and AF cells to mechanical loading, oxidative stress, inflammatory cytokines, and ECM degradation [91]. Monolayer cultures have demonstrated the role of MMPs and aggrecans in ECM degradation [92]. For example, antioxidant‐functionalized polymer capsules have been demonstrated to lower reactive oxygen species and inhibit the production of catabolic enzymes like MMP‐3 and ADAMTS‐5 in an IL‐1β‐induced NP cell model used to study oxidative stress‐mediated degeneration [81]. Similarly, IL‐1β‐induced NP inflammation models have used advanced biomaterial‐based in vitro systems, such as cross‐linked hyaluronic acid hydrogels, to control inflammatory signaling and reduce the expression of pain‐associated neurotrophins, including NGF and BDNF [82]. Despite their simplicity compared to natural disc structures, these models have proven crucial in targeting treatment and elucidating cellular behavior in degenerative diseases. However, traditional models cannot accurately replicate the avascular, hypoxic, and nutrient‐poor conditions specific to the IVD, limiting their ability to capture these interactions [41] effectively. These difficulties underscore the necessity of sophisticated in vitro models that can more accurately replicate the inflammatory and pain‐producing milieu of the degenerating disc.

Another tool for in vitro studies is explant culture, in which tissue is excised into small pieces and placed in culture dishes or flasks, allowing cells to migrate out and adhere to the surface [93]. For example, an NP explant model treated with chondroitinase ABC (chABC) mimics IVD degeneration by inducing loss of sulfated glycosaminoglycan (sGAG) and biomechanical impairment. The notochordal cell‐derived matrix promotes NP cell proliferation and matrix organization, highlighting its repair potential [83]. However, explant culture has limitations, including the absence of key immune cells, sourcing challenges, difficulty in maintaining optimal conditions, and a limited lifespan, which restricts long‐term studies. Additionally, it remains challenging to investigate the crosstalk mechanisms of mechanical loading to biochemical responses, such as inflammation and pain [42, 94].

### 4.2 Ex Vivo Models

Ex vivo models are crucial for studying IVD degeneration, as they maintain IVD cells in their natural 3D environment, preserve tissue integrity, and offer controlled conditions that closely mimic physiological conditions, making them more advantageous than ex vivo cultures [54, 95]. IVD degeneration can be induced by physical injury, mechanical loading, inflammatory cytokines, and enzymatic digestion [54]. Whole intervertebral disc organ culture systems utilizing bioreactors have been implemented to replicate both physiological and degenerative states via regulated mechanical loading and nutrient provision, revealing heightened cell mortality and catabolic activity under high‐frequency loading and restricted glucose availability [55, 96]. In these models, the efficacy of regenerative therapies, such as mesenchymal stem cell implantation, is influenced by the degenerative condition of the disc and the delivery vehicle, with improved anabolic responses observed in degenerative states when cells are administered within supportive biomaterials. A previous study showed that bovine IVDs cultured with intact cartilage end plates maintained cell viability and matrix integrity, whereas removing end plates or retaining vertebral bone led to excessive cell death due to impaired nutrient diffusion [84]. Physical disruption methods induce IVD degeneration more rapidly than other approaches [54]. A dynamically‐loaded whole organ culture model, such as a cavity defect model, was created via nucleotomy at the center of the endplate or through AF and cultured under simulated physiological loading (0.06 ± 0.02 MPa, 0.1 Hz, 6 h/day) for 6 days, demonstrated elevated compressive stress led to greater disc height loss, structural damage to the AF, protrusion of the inner AF and NP into the cavity, and reduced cellular viability [56]. Similarly, a bovine whole intervertebral disc organ culture model using partial nucleotomy through the endplate demonstrated that implantation of a polyurethane scaffold could restore disc height and compressive stiffness, while also downregulating catabolic gene expression, such as MMP13, in native disc tissue [97].

Inflammation‐based approaches have demonstrated typical degenerative features; for instance, IL‐1 treatment induces proteoglycan loss and increases catabolic enzyme expression, such as MMP‐13 and ADAMTS‐5, with more pronounced effects observed in miR‐146a knockout discs, highlighting its protective role against inflammation‐driven degeneration [98]. Additionally, chemokine‐mediated strategies have been investigated to augment endogenous repair, with stromal cell‐derived factor‐1 (SDF‐1) demonstrating the ability to facilitate mesenchymal stem cell migration into disc tissue, leading to elevated collagen type II expression and enhanced matrix production [99]. Similarly, TNF‐α induces an irreversible cellular transition towards catabolic processes [100], particularly under dynamic loading of certain conditions [101]. Models driven by proinflammatory cytokines, especially those utilising TNF‐α, are frequently used to replicate the inflammatory milieu linked to intervertebral disc degeneration. TNF‐α stimulation induces a catabolic shift in nucleus pulposus cells and in entire intervertebral disc organ cultures, marked by upregulation of matrix metalloproteinases and inflammatory mediators, as well as an increased release of nitric oxide, glycosaminoglycans, and cytokines, including IL‐6 and IL‐8 [79]. The disc's response to injury is strongly dependent on mechanical loading, with physiological loading preserving matrix integrity while unloaded conditions accelerate degeneration [102].

Studies have demonstrated that disc integrity and stiffness, which are compromised by trypsin injection or abnormal mechanical loading, can be partially restored by applying physiological loading [103]. Papain was used to induce loss of GAG and water content, resulting in increased severity of disc degeneration (Grades II, III, and IV) in an enzyme dose‐dependent manner [43]. Despite preserving the structural complexity, maintaining appropriate nutrient conditions will always be a challenge, especially with replicating the low‐glucose and hypoxic microenvironment of human IVD degeneration [104]. Furthermore, molecular analysis in ex vivo disc models remains technically challenging because of the low cellularity and high proteoglycan content of IVD tissue, which can interfere with RNA isolation and downstream gene expression analysis [105]. In addition, the roles of vasculature, innervation, and pain phenotype in IVD degeneration can only be studied in system‐level models and in vivo with intact circulatory and nervous systems, as ex vivo organ culture models do not provide this capability [59]. Addressing this issue is crucial for better replicating the human‐relevant IVD degenerative phenotype [57].

### 4.3 In Vivo Models

Animal models have been extensively used to study the pathophysiology of intervertebral disc degeneration, providing critical insights into its mechanisms and potential therapeutic approaches. The need for improved human‐representative models is highlighted by the fact that in vivo animal models provide a closer approximation of human disc degeneration. Rodent species, particularly mice and rats, are commonly employed because of their genetic tractability, cost‐effectiveness, and ability to mimic early degenerative changes through surgically induced changes, mechanical loading‐induced degeneration, or genetic modification [106, 107, 108]. Models of degeneration generated by mechanical loading simulate the biomechanical factors contributing to IVD degeneration by applying controlled abnormal stresses to the spine. In mouse tail models, static compression or dynamic loading via external fixation devices is commonly employed to induce degeneration, leading to diminished disc height, extracellular matrix breakdown, and increased apoptosis. Static compression applied to rat tails has been demonstrated to cause proteoglycan depletion, heightened matrix metalloproteinase production, and structural disorganization of the nucleus pulposus and annulus fibrosus [60, 109, 110]. Likewise, alterations in loading amplitude, duration, and frequency markedly affect the degree of degeneration, as excessive or high‐frequency loading fosters catabolic activity and cellular apoptosis [60]. These models are especially significant for investigating the mechanobiology of disc degeneration and its relationship with cellular responses.

Genetic models clarify the molecular causes of intervertebral disc degeneration by focusing on genes associated with extracellular matrix homeostasis and inflammatory control. For instance, biglycan‐deficient animals demonstrate expedited age‐related disc degeneration, characterized by structural disruption and matrix degradation, underscoring the significance of proteoglycan organization in preserving disc integrity [111]. Furthermore, transgenic and knockout models that focus on matrix‐degrading enzymes, such as ADAMTS5, have exhibited modified vulnerability to degeneration, thereby affirming their function in extracellular matrix catabolism [112]. Recently, spontaneous and strain‐specific mouse models have been established, exhibiting early‐onset degeneration defined by altered cellular phenotype and matrix homeostasis [107, 113]. These models are especially useful for clarifying gene‐specific roles in disc damage and for assessing focused treatment approaches.

In addition to controlled mechanical loading and genetic modification models, surgically induced injury models are widely used in vivo to replicate disc degeneration. Mechanical models disrupt or load the IVD, using degradative enzymes to mimic ECM degradation, and hybrid models combine both. These models are faster, more cost‐effective, offer better control, and raise fewer ethical concerns than in vivo models [40]. Mechanical methods for inducing IVD degeneration in vitro mainly fall into two categories: physical disruption and mechanical overloading. Models based on physical disruption induce degenerative processes by damaging key components of the IVD. This is often achieved by needle puncture of the AF and NP for annulotomy and nucleotomy, respectively [40, 44, 45, 46]. Studies have shown that AF puncture leads to cell death and loss of PGs and GAGs within 6 days. Similarly, murine IVDs exhibit reduced PGs (11–5 μg/mg) and collagen (3–1.9 μg/mg) 21 days after puncture with a 27G needle [40]. However, the needle height ratio influences degeneration, with 40% consistently inducing disc changes, while < 25% has minimal effects. In rats, a 27G needle (52% height) reduced stiffness (20%–60%) and increased neutral zone length (+130%), whereas a 33G needle (26% height) had mild effects (+20%). In sheep, a 27G needle (10% height) slightly reduced the torque range (−15%) without major stiffness changes [86]. Nucleotomy‐induced IVD injury demonstrated a microstructural degeneration classified as Thompson grade III, followed by increased galactosylation and sialylation, inflammation, nociceptive nerve fiber innervation (CGRP, GAP43), as well as upregulation of peripheral and spinal pain markers (c‐Fos and substance P), associated with pain behaviors including mechanical allodynia, and thermal hyperalgesia, providing a robust discogenic pain in rats [88, 89]. Another study demonstrated AF puncture‐induced lumbar IVD injury in rabbits, resulting in reduced disc height and tissue hydration, microstructural damage and increased inflammatory markers (IL‐1β and IL‐6), suggesting a suitable load‐bearing IVD model [87].

Despite the useful insights these models offer, they are limited by ethical considerations, especially regarding large mammals [114, 115]. In addition, their applicability to human IVD pathology is complicated by interspecies variation anatomically and biomechanically. The subjective experience of pain in animals and behavioral indicators may not accurately reflect the pain phenotype in humans [92]. It is difficult to accurately represent the unique environment and phenotype of human IVD degeneration, especially regarding chronic inflammation and pain [47]. These limitations highlight the need for complementary approaches, such as advanced in vitro systems, including organ‐on‐a‐chip technology, to bridge the translational gap in IVD degeneration research [116].

## 5 Rationale of Organ‐on‐a‐Chip to Miniaturize Intervertebral Disc

Organ‐on‐a‐chip technology enables the creation of human tissues and organ systems on microfluidic platforms, offering unprecedented insights into complex physiological and pathological microenvironments. Notably, the disc‐on‐a‐chip platform is an innovative tool for investigating IVD degeneration. It integrates microfluidics and a 3D ECM‐based system under pathological insults to accurately simulate the natural disc microenvironment. In contrast to conventional models, a key feature of the microfluidic system is essential for modulating nutrient transport, oxygen diffusion, and waste elimination, thereby simulating the intrinsic avascular and hypoxic environments of the disc. These fluidic systems facilitate the regulated administration of biochemical signals, growth factors, and therapeutic drugs, allowing the examination of real‐time cellular responses in physiologically relevant settings. Together, these strategies support the modular and reproducible design of disc‐on‐a‐chip models that replicate the key features of healthy and degenerated intervertebral discs. This technology facilitates real‐time observation of cell‐matrix interactions, pain signaling, and degeneration, making it an effective instrument for pharmacological evaluation.

The disc‐on‐a‐chip model facilitates cell‐matrix interactions, inflammatory cascades, neurogenic pain processes, and ECM regulation by integrating multiple cell types within a controlled micro‐engineered platform, enabling high accuracy in research. A pain‐relevant disc‐on‐a‐chip platform should ideally integrate disc cells, inflammatory stimuli, vascular interfaces, and sensory neurons, together with measurable functional outputs such as calcium signaling, neuronal firing, or neuropeptide release [117]. By connecting laboratory research with practical applications, the disc‐on‐a‐chip offers significant potential for personalized medicine and enhanced treatment approaches for IVD degeneration. They support high‐throughput, multiplexed, and parallelized assays, thereby increasing analytical capacity and efficiency. The miniaturized format enables faster analysis by reducing reaction and separation times. Microfluidic systems significantly reduce the consumption of reagents and overall analysis costs. Their portability makes them well‐suited for point‐of‐care diagnosis and therapeutic applications.

## 6 Strategies to Create the Heterogeneity of the IVD Anatomy and Composition

In the case of IVD degeneration, the organ‐on‐a‐chip platform offers physiologically relevant models that integrate the ECM‐based matrix and mechanical, chemical, and cellular components of the disc for modeling mechanisms underlying intervertebral disc degeneration [42]. Among its promising applications is mimicking the heterogeneous IVD structure, which comprises the NP and AF regions, by integrating microfluidics and ECM‐based hydrogel integrated with real‐time monitoring technologies. The system is typically structured with compartmentalized chambers that house NP and AF cells, facilitating controlled investigations into cell‐cell interactions. The choice of the cell sources is important to achieve targeted, physiologically relevant disc‐on‐a‐chip models. Human primary cells may resemble the native disc phenotype; however, they have restricted tissue availability, vary from donor to donor, differ in sex, age and the grade of degeneration, and change in the phenotype during in vitro cell culture expansion [12, 17, 18]. Animal primary cells provide better accessibility and reproducibility but differ from the native human IVD cells in terms of cellular composition and biological responses, leading to translational relevance [109, 110]. Similarly, iPSC‐derived neurons and/or immortalized macrophage cell lines might provide a reproducible cell source, but they might not accurately replicate the biological heterogeneity of their main counterparts [118, 119]. Thus, the selection of the cells is crucial for improving the physiological relevance of the model. These systems aim to replicate the 3D microenvironment of the intervertebral discs and then be used for disease modeling under biomechanical and pathological insults, providing valuable insights into disease mechanisms and therapeutic responses.

### 6.1 Microfluidic Disc‐on‐a‐Chip System

Microfluidic devices are commonly fabricated using microfabrication techniques such as 3D printing, photolithography, soft lithography, hot embossing, and injection molding, which define channel geometry, fluid control, and cellular microenvironment [63, 120]. Photolithography employs light‐mediated pattern transfer onto photoresist‐coated surfaces to create exact microscale features, whereas soft lithography often utilizes elastomeric polymers like PDMS, cast from master molds, to reproduce microchannel architectures [121]. Conversely, 3D printing facilitates rapid prototyping and the creation of complex three‐dimensional structures, such as perfusable channel networks and biomimetic tissue compartments [122]. Hot embossing and injection molding are predominantly employed for large‐scale and high‐throughput device manufacturing due to their reproducibility and efficiency in production [123]. Collectively, these fabrication techniques dictate channel layout, fluid dynamics, nutrient transport, and the biological microenvironment within microfluidic systems. Material selection and fabrication strategy directly influence device reproducibility, scalability, and physiological relevance. Materials such as PDMS, PMMA, cyclic olefin copolymer, hydrogels, and photocurable resins, together with fabrication strategies as mentioned above.

Two main mold strategies were employed in disc‐on‐a‐chip fabrication. First, in the positive mold method, printed channel structures are embedded in PDMS, cured, and removed to create hollow channels. Secondly, in the negative mold method, the inverse geometry is printed, and PDMS or hydrogel is cast into the mold to form flow paths or cell compartments [124]. Microfluidic devices are commonly fabricated using polydimethylsiloxane (PDMS) because of its biocompatibility, flexibility, optical transparency, and gas permeability, which make it ideal for simulating the hypoxic intervertebral disc environment [125]. However, PDMS usually absorbs small hydrophobic molecules, which alters the concentration of therapeutic compounds during drug screening and pharmacological studies, affecting experimental reproducibility and translational relevance [126]. Consequently, other alternative materials show lower molecular adsorption, better dimensional stability, resistance to chemicals, and high compatibility, making them more suitable for long‐term culture and other organ‐on‐a‐chip applications such as cyclic olefin copolymer (COC/COP), polymethyl methacrylate (PMMA), glass and thermoplastics [127, 128, 129]. Recently, 3D‐printed sacrificial mold techniques have surfaced as an advanced fabrication method for microfluidic disc‐on‐a‐chip systems. This process involves printing sacrificial materials that define the desired microchannel design before embedding them within PDMS, hydrogels, or other supporting matrices. Subsequent to curing or gelation, the sacrificial template is eliminated via dissolving or melting, resulting in perfusable hollow microchannels. Common sacrificial materials are carbohydrate glass, Pluronic F127, gelatin, agarose, and poly(vinyl alcohol) (PVA) because of their printability and facile removal under mild temperatures suitable for cell‐laden hydrogels [130, 131, 132, 133]. This concept facilitates the creation of intricate three‐dimensional channel networks with enhanced geometric accuracy and biomimetic nutrition delivery, surpassing traditional planar production methods. These fabrication approaches allow the integration of perfusion channels, NP chamber, and AF‐mimetic ring structures within a single platform.

The design of microchannels in disc‐on‐a‐chip systems fundamentally influences their capacity to mimic the avascular and nutrient‐deficient milieu of intervertebral discs. The continuous perfusion channels in the mouse disc‐on‐a‐chip system markedly enhanced nutrition delivery and waste elimination, maintaining NP and AF cell viability for up to 21 days, in contrast to conventional static culture [64]. In an “annulus‐on‐a‐chip” platform, custom channel shapes were engineered to emulate intricate in vivo strain patterns in the AF, facilitating physiologically relevant mechanical loading throughout the microchannel region [65]. A recent analysis highlighted that microchannel branching and array configuration can be customized to create precise nutritional gradients, shear stress conditions, and localized biochemical stimuli, simulating both healthy and degenerative disc states [42]. Although standardized channel designs are rare, these instances illustrate that differences in channel geometry, dimensions, and flow configurations are essential for the regulated supply of nutrients, oxygen, mechanical stresses, and inflammatory mediators in functional disc‐on‐a‐chip models. Physiological loading maintains extracellular matrix synthesis and cellular function, whereas abnormal or excessive loading disrupts tissue homeostasis and accelerates disc degeneration [42, 134, 135]. Mechanotransduction mechanisms involving mechanosensitive ion channels such as transient receptor potential cation channel 4 (TRPV4) and Piezo1, triggered by mechanical stimuli, activate downstream inflammatory signaling pathways such as NF‐κB, leading to increased expression of matrix metalloproteinases (MMPs), pro‐inflammatory cytokines, and extracellular matrix degradation [136, 137]. Thus, it is important to integrate controllable compressive, tensile, hydrostatic, or shear loading into future disc‐on‐a‐chip platforms to recapitulate the native biomechanical microenvironment and its influence on disease progression [42, 65].

To enhance microchannel design, computer simulations such as computational fluid dynamics (CFD) and finite element analysis (FEA) are used to optimize disc‐on‐a‐chip platforms. These models can predict the impact of microchannel geometry on fluid dynamics, nutrient diffusion, shear stress, and mechanical strain, enabling researchers to evaluate design modifications in silico before production. Software such as COMSOL Multiphysics, ANSYS Fluent, and OpenFOAM are frequently employed in microfluidic and tissue engineering applications because of their ability to simulate coupled transport and mechanical processes. CFD models in COMSOL can model the development of oxygen and glucose gradients inside the NP region, emulating the avascular conditions of the native disc. This facilitates the development of channel shapes that sustain cell viability by allowing sufficient nutritional transport while maintaining the hypoxic conditions typical of the inner disc [66]. Furthermore, recent research employing specimen‐specific FEA models of intervertebral discs validated through in vitro imaging illustrates how predictive modeling of disc bulging and load distribution can inform precise disc‐inspired designs in microfluidic systems [138]. Even with these advances, it is still difficult to integrate all these engineering parameters into a reproducible and robust platform. The optimization of one design parameter based on the material selection, channel geometry and hydrogel characteristics may affect oxygen diffusion, nutrition delivery, waste elimination or mechanical stimulation within the device as they are interconnected. Thus, both experimental validation and computational simulation are essential to establish physiologically meaningful and reproducible design parameters for disc‐on‐a‐chip systems.

### 6.2 Hydrogel Disc‐on‐a‐Chip System to Mimic the Disc Microenvironment

Mimicking the disc microenvironment requires the integration of several key features to accurately recapitulate the native tissue conditions. First, establishing a 3D ECM niche is critical, as it provides structural and biochemical support that influences cell behavior and matrix remodeling. Replicating the disc microenvironment entails reproducing essential biochemical and biophysical signals, such as a customized 3D extracellular matrix, hypoxia, nutritional gradients, and mechanical loading, which govern cellular behavior, including stem cell survival, self‐renewal, proliferation, and differentiation [139]. To ensure that the engineered platform accurately replicates the native disc microenvironment and maintains native nucleus pulposus and annulus fibrosus cell phenotypes, the successful implementation of these microenvironmental cues should also be experimentally validated using oxygen and pH sensing, computational or fluorescent tracer‐based gradient verification, and metabolic monitoring, such as glucose consumption and lactate production [67, 140]. Given that many cells, particularly stem cells, depend on distinct niche‐specific signals, precisely replicating these conditions is essential for successful regeneration of the intervertebral disc. This niche must closely resemble the natural extracellular matrix composition found in intervertebral discs, ensuring that cells receive appropriate signals to maintain their function.

ECM‐based Hydrogels are commonly used to recapitulate the composition and functional properties of the AF and NP, providing swelling pressure, effectively sustaining mechanical stresses, preventing leakage, supporting cell proliferation and survival, and inducing anabolism [141]. Hydrogels are particularly favored for their tunable mechanical properties and ability to sustain cell viability, closely simulating the hydrated environment of the native disc. The choice of biomaterial is critical for designing a hydrogel‐based disc‐on‐a‐chip system. To further enhance the composition relevance of the NP, biomaterials such as HA and collagen hydrogels could be incorporated into the microfluidic system to create a 3D environment that mimics the ECM component of the native disc, thereby maintaining cell phenotype in a 3D niche. A recent study developed a hybrid hydrogel composed of decellularized NP matrix and chitosan, combined with GDF5‐releasing microspheres and NP stem cells, which successfully promoted NP‐like differentiation in vitro and prevented disc degeneration in a rat model more effectively than BMSCs alone [142]. A study demonstrated that an engineered methacrylated hyaluronic acid hydrogel that mimics the interface between primary sensory nerves and the intervertebral disc, with hydrogel containing chondroitin‐4‐sulfate, showed a neuroinhibitory effect compared to chondroitin‐6‐sulfate, demonstrating a 3D in vitro disc innervation model of the IVD [48]. The HA/Collagen type II hydrogel alleviated pain behavior, reduced sensory hyperinnervation, and restored disc hydration in a rat tail degeneration model, highlighting its dual regenerative and analgesic potential [88].

Additionally, maintaining the phenotypic markers of NP and AF is essential. For example, collagen‐based hydrogels have shown the ability to sustain NP cell phenotype, including prolonged type II collagen and proteoglycans expression and maintained the rounded cell morphology typical of native NP cells [49]. NP cells exhibit distinct markers, such as high expression of aggrecan and type II collagen, whereas AF cells express markers such as type I collagen [143, 144]. Our previous study demonstrated that the HA/COLII hydrogel was non‐cytotoxic, which provides an optimal stability, swelling capacity, and degradability, effectively mimicking the NP microenvironment to guide hWJ‐MSCs differentiation into NP‐like cells and maintain their phenotype (SOX9) [145]. Likewise, biomimetic hydrogel and scaffold systems for annulus fibrosus repair facilitate extracellular matrix organization, mechanical integrity, and fibrocartilaginous features, underscoring the significance of matrix‐mimicking materials in maintaining annulus fibrosus phenotype and function [146, 147]. The use of biomimetic hydrogel‐based scaffolds and extracellular matrix components guarantees optimal cell adhesion, proliferation, and differentiation, promoting the development of a three‐dimensional environment that closely mimics natural disc tissue. Hydrogels, such as collagen and gelatin methacryloyl (GelMA), have been widely employed in disc‐on‐a‐chip models to support NP and AF cell viability, promote extracellular matrix synthesis, and maintain a disc‐like phenotype [148].

Reproduction of hypoxia is crucial because the native NP is avascular and operates under low oxygen tension, about 1%–5%, which is necessary for its specific metabolic profile [149, 150]. Hypoxia increased autophagic activity by increasing the expression of beclin‐1 and LC3‐II/LC3‐I ratio, activating SIRT1, while concurrently inhibiting apoptotic signaling through the downregulation of Bax and caspase‐3 expression [149]. Besides affecting autophagy and apoptosis, hypoxia also influences post‐translational regulatory pathways in intervertebral disc cells. A previous study indicated that both NP and AF cells preserved viability under hypoxic circumstances while displaying modified expression of SUMOylation‐related proteins, such as SUMO‐1, SUMO‐2/3, SAE1/2, UBC9, and SENP1 [151]. Moreover, the inhibition of SENP1 diminished HIF‐1α transcriptional activity, indicating that SUMOylation pathways play a role in hypoxia adaptation and the regulation of cellular homeostasis in disc cells. Hypoxia‐inducible factor‐1α (HIF‐1α) in NP cells helps regulate glycolytic metabolism and enhances the aggrecan and collagen type II expression in the hypoxic environment [152]. Another study also confirmed that the NP cell phenotype was enhanced in hypoxic conditions, with greater production of sulfate glycosaminoglycans and collagen type II. However, AF cells were not primarily affected by this condition [153].

Nutrient gradients, including variations in glucose and pH, must be established to simulate the in vivo metabolic environment. Because of the avascular nature of the IVD, nutrient diffusion occurs through the cartilage endplate and AF, which creates metabolite gradients within the NP region [154]. The most critical nutrient for disc survival is glucose, which makes it crucial in increasing the NP cell viability, particularly under acidic conditions. In contrast, it was observed that glucose availability is more critical than oxygen for maintaining viability due to IVD cells, particularly NP cells, showing tolerance under severe hypoxia [155]. Nutrient transport can also be impaired under several conditions, particularly degeneration and endplate calcification, which will promote acidification within the disc microenvironment [156]. Acidic pH conditions typical of degenerative discs have been demonstrated to elicit a catabolic and proinflammatory phenotype in NP cells, which is characterized by elevated production of IL‐6, IL‐1β, NGF, BDNF, and matrix‐degrading enzymes like ADAMTS‐4 and MMP‐3 [136].

Additionally, biomechanical actuation applies compressive, tensile, and shear forces to simulate the physical loads experienced by the disc in the body, providing insights into how mechanical stress influences degeneration [157]. Incorporating mechanical loading, such as cyclic compression, shear stress, and tensile forces, allows for the study of biomechanical influences on cell behavior and matrix integrity [50]. Under physiological conditions, regulated dynamic loading facilitates nutrition transport and anabolic matrix formation, while excessive or prolonged mechanical loading induces catabolic signalling, oxidative stress, apoptosis, and extracellular matrix breakdown [134]. Abnormal mechanical loading activates mechanotransduction pathways that involve NF‐κB, ROS, MAPK, Piezo1, and the NLRP3 inflammasome, resulting in heightened production of inflammatory cytokines and cellular responses linked with degeneration [135, 157].

### 6.3 Replicating Lamellae of the Annulus Fibrosus

Replicating the angle‐ply architecture of the AF, where collagen fibers alternate in orientation between ± 30° and ± 60°, is critical to mimicking the anisotropic mechanics of the disc. Electrospinning is widely used to fabricate aligned fibrous scaffolds by layering fiber mats with alternating orientations. Materials such as polycaprolactone (PCL), collagen, and silk fibroin have been shown to guide AF cell alignment and enhance collagen type I deposition [158]. Three‐dimensional bioprinting allows the programmable deposition of materials in specific orientations, enabling layer‐by‐layer control of filament angles to mimic concentric AF lamellae. These bio‐printed constructs support region‐specific architecture and integration with NP‐like cores, promoting appropriate matrix organization and mechanical gradients [159, 160]. Fiber‐reinforced hydrogels combine the mechanical anisotropy of aligned fibers with the cell‐permissive environment of soft hydrogels, such as collagen or GelMA. This approach improves the tensile strength while supporting AF cell alignment and matrix production [161, 162]. Microfluidic‐based platforms can use shear flow, magnetic fields, or patterned microchannels to align fibers during gelation. These methods offer microscale control but typically replicate only single‐angle alignments, limiting their ability to fully recreate multilayered AF structures [163].

### 6.4 Multicellular Integration for Functional Disc‐on‐a‐Chip Model

Integrating multiple cell types is essential for developing a functional disc‐on‐a‐chip that accurately mimics the complex microenvironment of the IVDs. A co‐culture system of NP and AF cells can effectively model the interactions between these two cell types, which is vital for understanding disc physiology and degeneration progression. This system could integrate NP and AF cells to preserve the structural and biochemical properties of the disc while facilitating dynamic interactions that promote degeneration. Human AF cells have been successfully cultured in microfluidic platforms under inflammatory conditions [68], where macrophage‐derived cytokines induce inflammatory mediators upregulation, such as IL‐6, IL‐8, and TNF‐α and matrix‐degrading enzymes, including MMP‐1, alongside activation of NF‐κB signaling, reflecting a degenerative and catabolic phenotype. At the same time, human NP cells cultured in a microfluidic system [164] showed an enhanced response of inflammation, such as IL‐8 and IL‐6, as well as ECM degradation (MMP‐1 and MMP‐3), while being co‐cultured with macrophages. To replicate more accurately the inflammatory circumstances linked to IVD degeneration, immune cells, including macrophages and T cells, may be incorporated to investigate their function in chronic inflammation and extracellular matrix degradation. THP‐1 monocytes have been integrated into disc‐on‐a‐chip systems to study IL‐1β‐induced chemotactic migration and differentiation into macrophages, highlighting their role in the degenerative microenvironment [69, 82]. The incorporation of nociceptive neurons is essential for understanding the mechanisms underlying discogenic pain, as these neurons engage with inflammatory mediators and enhance pain sensitivity. Recent studies have utilized induced pluripotent stem cell (iPSC)‐derived nociceptors co‐cultured with NP cells in microfluidic platforms to investigate pain signaling and neuroinflammation [51].

## 7 Disc‐on‐a‐Chip to Model Intervertebral Disc Degeneration

IVD degeneration induces chronic discogenic pain through a complex network of molecular and cellular mechanisms involving ECM degradation, inflammation, angiogenesis, innervation, and sensory sensitization [14].

### 7.1 Modulation of Matrix Degradation

The ECMs, such as collagen, proteoglycans, and elastin, are abundantly found in the IVD, mainly to provide structural support to the disc. During IVD degeneration, the release of pro‐inflammatory factors (IL‐1β and TNF‐α) triggers the upregulation of MMPs, which results in the degradation of collagen and proteoglycans that are pivotal to maintaining the tissue hydration and load‐bearing capacity [165, 166]. The cumulative effect of matrix degradation, rather than the activity of individual proteases, is most pertinent to disease progression. The gradual depletion of ECM components diminishes tissue stiffness, undermines biomechanical stability, and enhances matrix permeability, thus promoting vascular and neuronal ingrowth into the avascular disc region [167, 168].

From a disc‐on‐a‐chip viewpoint, extended ECM degradation constitutes a vital design factor that can be simulated by the controlled adjustment of hydrogel composition, stiffness, and degradation kinetics. For example, a 3D self‐assembled collagen hydrogel has been used to study the response of NP and AF cells under inflammatory stimulation, demonstrating downregulation of key ECM markers, including collagen and aggrecan, thereby revealing inflammatory and ECM degradation crosstalk in the diseased model [169]. Hydrogel‐based systems utilizing GelMA or alginate matrices exhibit that enzymatic or cytokine‐mediated degradation results in diminished GAG levels and heightened matrix metalloproteinase (MMP) expression, mirroring essential characteristics of disc degeneration [170, 171, 172]. These findings underscore the capacity of hydrogel characteristics to modulate catabolic activity and matrix remodeling in disc cells directly.

### 7.2 Modeling Inflammation

Pro‐inflammatory mediators play a key role in both initiating inflammation and driving the progression of IVD degeneration [173, 174]. In degenerated IVDs, inflammation driven by immune cells is amplified, and their degradation products contribute to a decline in NP cell numbers and ECM degradation [91]. The degenerative process degrades the ECM and depletes hydrophilic molecules, causing structural and biomechanical changes in the IVD. This promotes inflammation, nerve ingrowth, and the release of pain factors [91, 175]. Inflammatory stimulation by mechanical stress, injury, or aging initiates the release of pro‐inflammatory cytokines, including IL‐1β and TNF‐α [176] through NF‐κB signaling pathway as a primary signaling [177]. NF‐κB activation integrates inflammatory cues to drive coordinated downstream responses, including upregulation of matrix‐degrading enzymes such as MMPs, pro‐angiogenic factors, for example, VEGF, and neurotrophic factors [178, 179, 180], which leads to the degradation of the ECM and the formation of new blood vessels within the disc known as angiogenesis [181, 182]. Neurotrophic factors such as NGF and BDNF induce innervation and sensory sensitization by increasing the excitability of nociceptors [183]. This leads to a lower threshold for pain processing and contributes to both peripheral and central sensitization, intensifying the pain signaling [175]. Collectively, these key pathways contribute to the progression and persistence of chronic pain in IVD degeneration [184].

**FIGURE 3:**
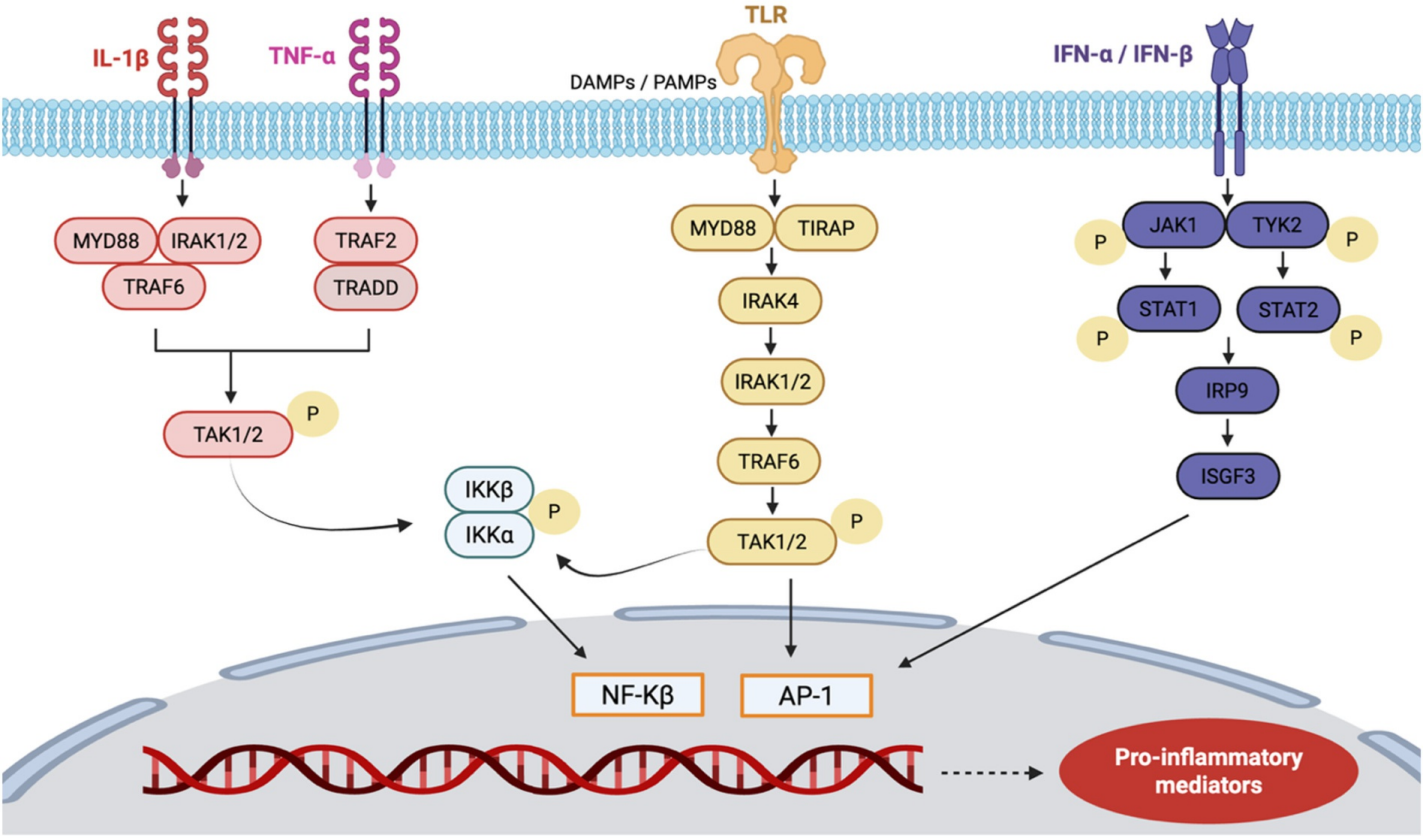
Inflammatory signaling integration and downstream pathological outputs relevant to intervertebral disc degeneration and bioactive disc‐on‐a‐chip systems. Cytokine, TLR, and type I IFN signaling pathways activate inflammatory transcriptional regulators, including NF‐κB, AP‐1, and ISGF3, leading to the production of pro‐inflammatory mediators in disc cells. Persistent inflammatory signaling contributes to angiogenesis, matrix remodeling, neurogenic sensitization, nociceptor activation, and discogenic pain. These processes represent key biological mechanisms and measurable functional outputs relevant to the design of bioactive disc‐on‐a‐chip platforms. Created in https://BioRender.com.

From a disc‐on‐a‐chip perspective, NF‐κB functions less as a molecular pathway of interest but more as a systems‐level integrator whose downstream outputs represent measurable and controllable features within a microphysiological model. The integration of cytokine signaling, Toll‐like receptor (TLR) activation, and type I interferon (IFN) signaling pathways through key inflammatory transcriptional regulators, including NF‐κB and AP‐1, leads to the production of pro‐inflammatory mediators as summarized in Figure 3. Sustained inflammatory activation leads to a downstream of pathological processes that include inflammation, angiogenesis, matrix remodeling, neurogenic sensitization, nociceptor activation, and ultimately discogenic pain [185]. By precisely regulating fluid flow and chemical gradients, these chips enable the modeling of inflammatory cascades, allowing researchers to simulate the sequential release of chemokines and cytokines, such as TNF‐α, IL‐1β, and IL‐6. This setup facilitates the study of cellular interactions, enabling detailed observations of how individual cells communicate, respond to pro‐inflammatory signals, and contribute to the progression of inflammation. A cutting‐edge microfluidic approach for immune cell isolation and functional assessment was innovated. Monocytes play a primary role in the immune system and retain their biological functionality, making the platform suitable for downstream applications such as immune response studies and cytokine profiling [69]. The high‐purity monocyte isolation provided by this device can enhance inflammation models by improving the accuracy of immune cell interactions in inflammation studies by providing a reliable source of primary monocytes for studying immune responses and enabling real‐time monitoring of inflammatory reactions, such as cytokine release and immune cell migration.

**FIGURE 4:**
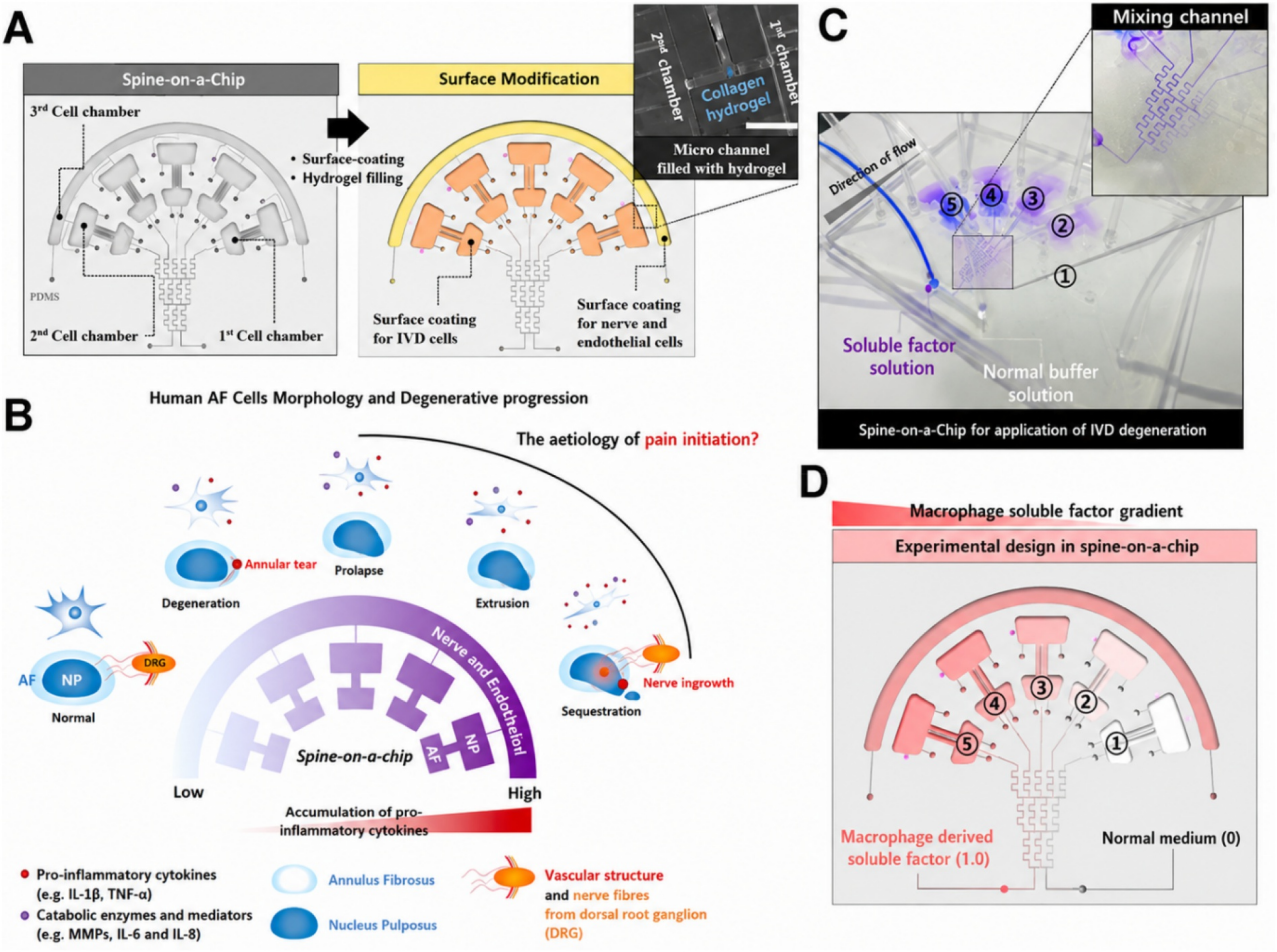
Inflammatory‐based IVD‐on‐a‐chip models. (A) The device is composed of three chambers designed to culture AF, NP, and nerve or endothelial cells (Chambers 1, 2, and 3, respectively) and comprises a gradient generator. Chambers 1, 2, and 3 were coated with fibronectin and poly‐D‐lysine to facilitate cellular adhesion, and the small channels connecting Chambers 2 and 3 were filled with a collagen‐based hydrogel. (B) Conceptual use of the devices. Different disease states are mimicked in the model as the cytokine concentration in the chambers increases (indicated by the purple color). (C) Functional validation of the model. The gradient generator allows for the establishment of a concentration gradient in different culture chambers. (D) Design of an experiment investigating the effects of a gradient of macrophage‐derived factors on IVD cell behavior. Reproduced under terms of the CC‐BY license [70]. Copyright 2017, The Authors, published by AIP Publishing.

These platforms provide critical insights into cytokine dynamics and immune cell recruitment. Real‐time monitoring within the chip allows the observation of temporal cytokine release profiles and the spatial organization of immune cells as they migrate toward inflammation sites [186]. This detailed mapping of immune cell trafficking and activation offers a deeper understanding of the cellular mechanisms underlying inflammatory responses. A two‐channel apparatus was introduced by Hwang et al. to co‐culture annulus fibrosus cells and subject them to proinflammatory factor gradients [70]. The application of this biological mechanism within the microfluidic platforms has enabled the development of inflammatory disc‐on‐a‐chip systems that more closely mimic the degenerative microenvironment (Figure 4). This enabled us to investigate how annulus cells react to inflammatory mediators through dose‐dependent catabolism. A three‐channel microfluidic system was recently created by Hwang et al. to investigate the chemotactic invasion of IVD cells under inflammatory IVD conditions [52]. Additional research has developed multichannel microfluidic devices to cultivate IVD cells and examine the effects of electrical stimulation on annulus cells [68] and flow rates (lower than those linked to physiological motion) [187]. Ultimately, inflammation‐on‐a‐chip models serve as powerful tools for deciphering the complex interplay between cytokine signaling and immune cell behavior, advancing the development of targeted anti‐inflammatory therapies.

### 7.3 Modeling Angiogenesis and Innervation

**FIGURE 5:**
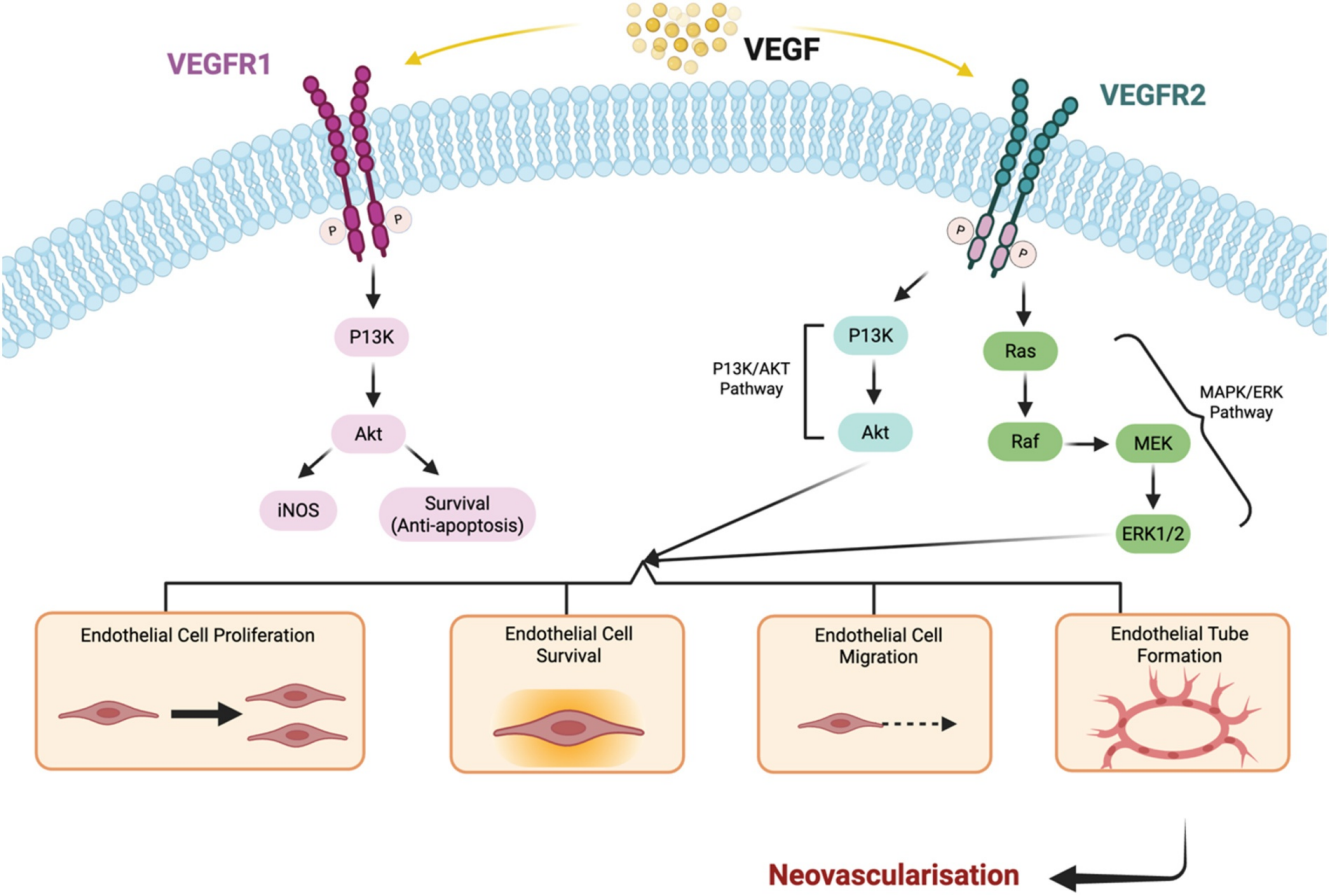
VEGF‐mediated angiogenesis and pathological neovascularization in intervertebral disc degeneration. Disc degeneration promotes VEGF upregulation and activation of VEGF receptors, stimulating downstream PI3K/Akt, Ras/Raf/MEK/ERK, and MAPK/ERK signaling pathways in endothelial cells. These signaling cascades enhance endothelial cell proliferation, survival, migration, and tube formation, leading to pathological angiogenesis and neurovascular ingrowth within degenerated intervertebral discs. Created in https://BioRender.com.

In healthy discs, NP has been shown to naturally suppress endothelial cell migration by releasing an anti‐angiogenic ECM composed primarily of proteoglycans [91]. However, degeneration leads to aberrant innervation of nociceptive nerve fibers into the inner AF and NP, which is a primary contributor to LBP [188, 189]. Degradation of the ECM enables the formation of new blood vessels, also known as angiogenesis [178]. Among the mediators involved in disc degeneration, VEGF, a pro‐angiogenic factor, is upregulated in degenerated discs, primarily through NF‐κB activation [190]. The downstream signaling pathways activated by VEGF are illustrated in Figure 5. VEGF binds to its receptors, VEGFR‐1 and VEGFR‐2, on endothelial cells, stimulating their migration, proliferation, and survival, resulting in the formation of new blood vessels within the avascular IVD [178]. Degradation of ECM by MMPs allows endothelial cells to infiltrate deeper into the disc tissue. The invasion of blood vessels into the degenerated disc is often accompanied by the infiltration of sensory nerve fibers, further contributing to peripheral sensitization and chronic pain [166]. In the degenerated disc, angiogenesis leads to pathological vascularization and supports neurovascular ingrowth, where sensory nerves infiltrate along vascular structures. Chronic inflammation induces the release of neurotrophic factors. NGF stimulates axonal sprouting and the production of neuropeptides, including substance P and CGRP [191], amplifying pain signaling and inflammation [192].

Additionally, innervation is limited to the outer third layer of the AF, while NP remains aneural. But, during degeneration, the structural disruption of AF, which includes the tears and fissures, will allow the ingrowth of nociceptive fibers into the deeper regions of the disc [14]. This process is predominantly influenced by inflammation, wherein pro‐inflammatory cytokines such as interleukin‐1β (IL‐1β) and tumor necrosis factor‐α (TNF‐α) enhance the synthesis of neurotrophins, including nerve growth factor (NGF) and brain‐derived neurotrophic factor (BDNF), which facilitate the extension of nerve fibers into the inner annulus fibrosus and nucleus pulposus [31]. The ingrowing nerve fibers are primarily nociceptive, exhibiting markers such as calcitonin gene‐related peptide (CGRP) and substance P, contributing to pain transmission. Moreover, degenerative discs demonstrate heightened expression of growth‐associated protein 43 (GAP43), signifying active axonal growth and neuronal plasticity [193]. This neurovascular ingrowth is frequently associated with neovascularization, which enhances nerve infiltration into typically aneural areas [194]. These modifications collectively lead to the sensitization of nociceptors and are closely linked to the onset of discogenic pain [31].

**FIGURE 6:**
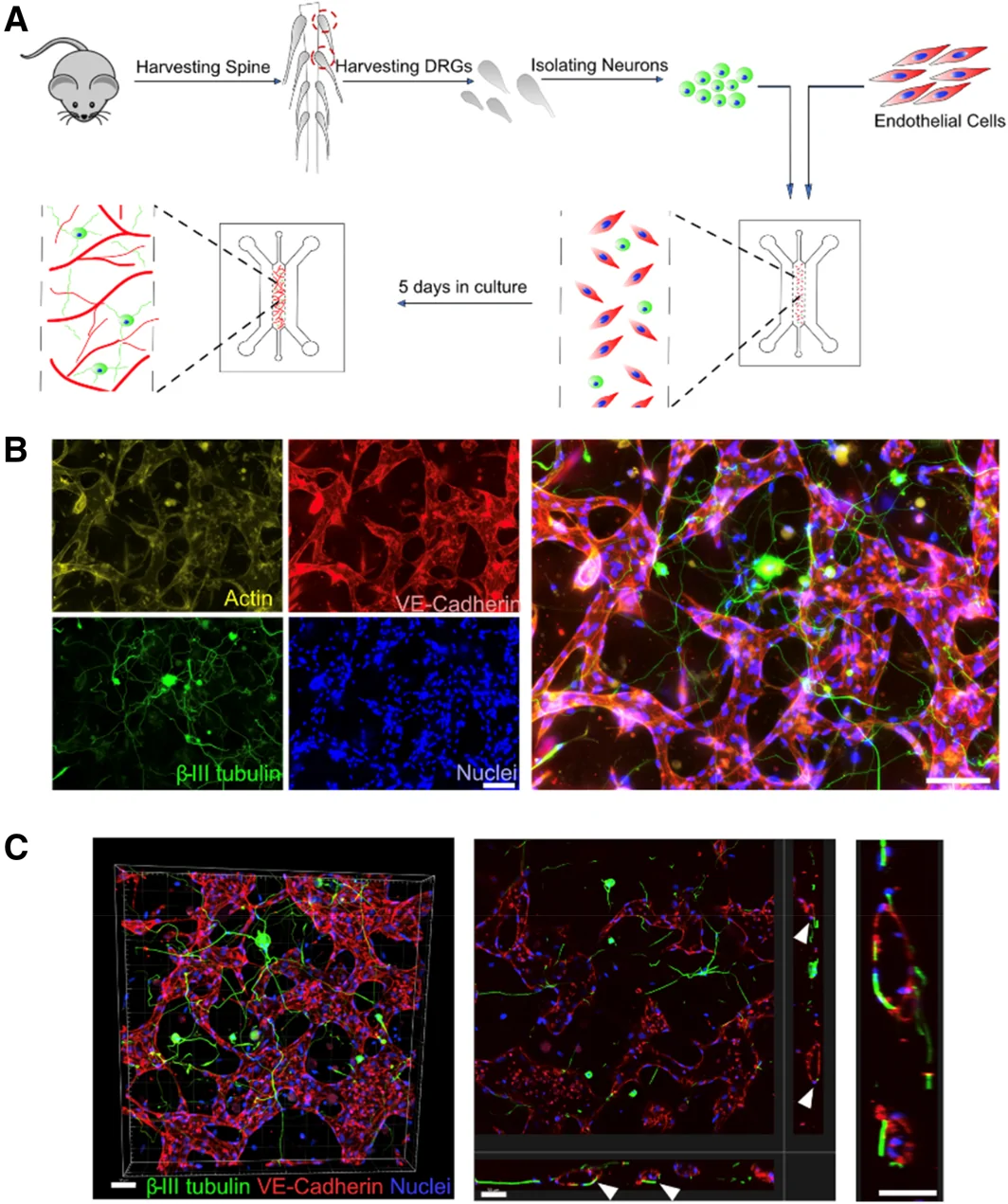
Development of an innervated vasculature model. (A) Schematic depiction of cell isolation and the process of generating innervated vasculature tissue by co‐culturing DRG neurons and endothelial cells (HUVECs). (B) Characterization of the innervated vascular network using immunostaining for endothelial (VE‐cadherin) and neuronal markers (β‐III tubulin). (C) 3D *z*‐stack images of the tissue showing direct contact between the vasculature and neurites. White arrows indicate the co‐localisation of β‐III tubulin and VE‐cadherin. Scale: 100 μm in (B) and 50 μm in (C). Reproduced under terms of the CC‐BY license [71]. Copyright 2023, The Authors, published by IOP Publishing.

As disc‐on‐a‐chip progresses, incorporating neurovascular interactions has become increasingly important for modeling painful intervertebral disc degeneration. A study demonstrated that the presence of vascularization significantly enhances nociceptive responses in sensory neurons, suggesting a crucial role for endothelial‐sensory neuron interactions in pain sensitization, as shown in Figure 6 [71]. Using an innervated vasculature‐on‐a‐chip platform, the researchers found that DRG neurons exhibited increased sensitivity to capsaicin, a transient receptor potential vanilloid 1 (TRPV1) agonist, in the presence of the microvasculature. This effect was associated with elevated expression of TRPV1, a key nociceptor involved in nociception, indicating that vascularization influences neuronal excitability. Furthermore, the platform was used to model acidosis‐induced pain by reducing the pH of the vascular perfusate, resulting in a dose‐dependent increase in sensory neuron activation. Comparable organ‐on‐a‐chip technologies have been extensively utilized to simulate angiogenesis and neurovascular interactions in various clinical situations. Vascularized microfluidic models have been created to investigate angiogenic sprouting and endothelial network creation under regulated flow conditions [72, 195].

### 7.4 Modeling Nociception

**FIGURE 7:**
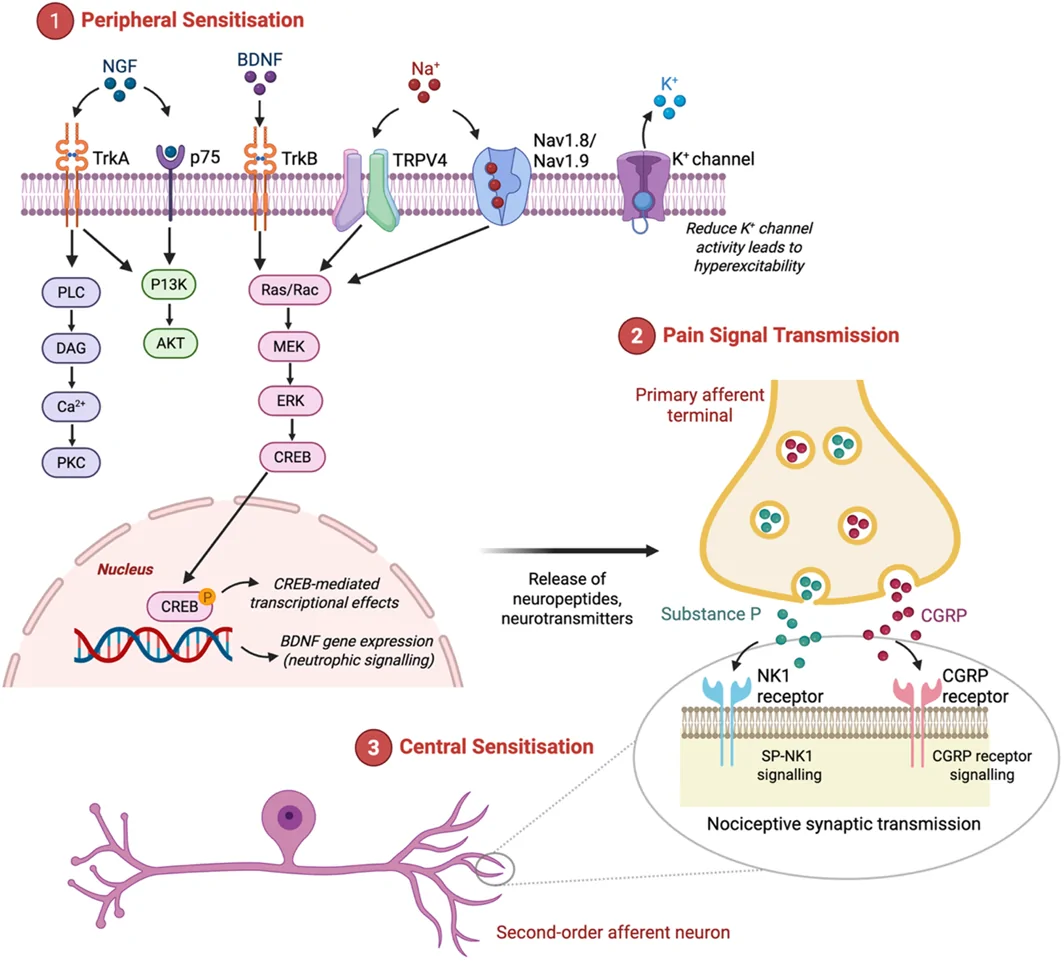
Peripheral nociceptor sensitization and central sensitization in pain signaling. NGF, BDNF, IL‐1β, and TNFα activate PI3K/Akt, PLC/PKC, and MAPK/ERK/CREB signaling pathways, promoting transcriptional changes, TRPV4 and voltage‐gated sodium channels contributing to neuronal transduction. Reduced K^+^ channel activity further enhances neuronal excitability, resulting in pain signal transmission up to the spinal level. An increased release of substance P and CGRP from primary afferent neurons results in nociceptive synaptic transmission that contributes to central sensitization in the dorsal horn of the spinal cord. Created in https://BioRender.com.

Chronic inflammation and neurovascular proliferation ultimately enhance peripheral nociceptor sensitization and central pain processing. These interconnected mechanisms are summarized in Figure 7. In IVD degeneration, neurotrophic factors such as NGF and BDNF are significantly upregulated within degenerated discs [181]. The binding of NGF to TrkA activates several intracellular signaling pathways, including MAPK/ERK, PI3K/Akt, and PLCγ, which lead to the transcription of genes that increase the expression of ion channels like transient receptor potential vanilloid 1 (TRPV1) and voltage‐gated sodium channels such as NaV_1.7/1.8_ [118], lowering the activation threshold of nociceptors, and enhancing neuronal excitability [118]. Inflammatory cytokines, such as TNF‐α and IL‐1β, increase nociceptor sensitization [184], while acidic and hypoxic environments enhance the activity of ion channels and maintain nociceptor activation [196]. This ongoing peripheral sensitization is characterized by enhanced responsiveness and spontaneous firing of nociceptors, which send amplified pain signals to the dorsal horn of the spinal cord [197].

Current evidence indicates that transient receptor potential vanilloid 4 (TRPV4) may have a role in intervertebral disc (IVD) degeneration and discogenic pain due to its function as a mechanosensitive and osmosensitive calcium‐permeable ion channel [198]. In contrast to TRPV1, which is mostly linked to nociceptive neurons and inflammatory or acidic stimuli, TRPV4 is engaged in detecting mechanical loading and osmotic variations within disc cells, both of which are modified during degeneration [198, 199]. TRPV4 has been demonstrated to modulate inflammatory cytokine networks under both static and dynamic compression, including the modulation of IL‐6 family cytokines and chemokines linked to immune cell recruitment [137]. The activation of TRPV4 can enhance the levels of IL‐6, IL‐11, and leukemia inhibitory factor (LIF), while concurrently diminishing the synthesis of chemokines associated with T‐cell and monocyte recruitment, suggesting a multifaceted role in preserving immunological homeostasis within the disc.

Central sensitization occurs when BDNF, released from both primary afferent neurons and activated microglia, binds to the TrkB receptor on dorsal horn neurons, which triggers intracellular signaling cascades, including MAPK/ERK and PI3K/Akt, which potentiate excitatory synaptic transmission [200]. The downregulation of inhibitory neurotransmitters, such as GABA and glycine, in the spinal cord further exacerbates dorsal horn hyperexcitability, leading to central sensitization [201]. Clinically, this manifests as allodynia and hyperalgesia, extending pain sensitivity beyond the original injury site and contributing to the persistence of chronic pain in IVD degeneration [202]. Thus, the upregulation of NGF and BDNF in IVD degeneration leads to both peripheral and central sensitization, creating a self‐perpetuating cycle of pain that is driven by neurogenic factors, inflammation, and matrix remodeling, which highlights the critical role of neurotrophic factors in the development and maintenance of chronic discogenic pain.

Disc‐on‐a‐chip model leverages engineered microfluidic platforms that integrate sensory neurons, DRG cells, and inflammatory mediators to recreate the nociceptive signaling cascades observed in IVD degeneration. These systems provide a controlled environment to study the molecular and electrophysiological responses of neurons to pro‐inflammatory cytokines such as TNF‐α, IL‐1β, and IL‐6, which are known to contribute to discogenic pain [203, 204]. Importantly, disc‐on‐a‐chip platforms are designed to model peripheral nociceptive mechanisms [42] rather than the multidimensional experience of pain [205]. Ideally, pain relevance should be evaluated at multiple levels, including molecular biomarkers (e.g., NGF, BDNF, TRPV1, and IL‐6) [176], cellular responses (e.g., neurite extension and neuroimmune interactions) [52, 71] and functional neuronal readouts (e.g., calcium imaging, electrophysiology, or neuropeptide release) [53], with regard to in vivo or clinical observations where relevant. Hwang et al. established one of the earliest models for investigating gradient‐based mechanisms in inflamed IVD cells. However, it has the potential to study the basic mechanisms of pain with the integration of neuron and vascular components through NGF or VEGF gradients [206]. A recent study indicated a relationship between human AF cells and endothelial cells, which are the target cells of IL‐8 and predominantly facilitate angiogenesis. These interactions provoke irritation of neurons originating from the DRG via the synthesis of nerve innervation mediators, which are regarded as the primary instigators of discogenic pain [207]. Furthermore, co‐culture models of immune cells and fibrocartilaginous disc cells can be employed to study neuro‐immune interactions and their role in nociception.

Neuroinflammatory crosstalk is a key contributor to IVD degeneration‐associated pain. Disc‐on‐a‐chip models could facilitate real‐time monitoring of the sensitization of nociceptors by inflammatory mediators, leading to chronic pain. These models allow researchers to examine the interaction between inflammatory cytokines and neuronal excitability, evaluate the function of glial cell activation and neuroimmune signaling in chronic pain, and identify prospective pharmacological targets for the modulation of neuro‐inflammatory responses. By mimicking in vivo‐like conditions, the pain‐on‐a‐chip technology provides a more physiologically relevant approach than traditional in vitro models. This enhances drug discovery efforts aimed at developing novel analgesics and anti‐inflammatory therapies for IVD degeneration.

## 8 Opportunities for Precision Disease Modeling

Building on the structural and biological design strategies described above, disc‐on‐a‐chip systems offer new opportunities for precision disease modeling, mechanistic discovery, and translational applications.

### 8.1 Studying Molecular Mechanisms Underlying Aging IVD Degeneration

One of the key insights these systems offer is into the senescence‐associated secretory phenotype (SASP) and the role of inflammation in disc degeneration. By simulating the pathological microenvironment of the disc, organ‐on‐a‐chip models enable us to study the interactions between senescent cells and neuroinflammatory mediators in modulating nociceptive circuitry, providing valuable data on how these factors drive degeneration and nociception in aging IVD degeneration. Previous studies on human disc tissue have demonstrated a higher prevalence of SA‐β‐gal‐positive senescent cells in herniated discs than in non‐herniated discs [208]. Likewise, SA‐β‐gal‐positive cells were detected in aging and degenerating discs of both humans and sand rats [209]. Previous studies have shown that it is important to investigate senescence using the organ‐on‐a‐chip approach.

### 8.2 Preclinical Drug Screening

In preclinical drug screening, organ‐on‐a‐chip systems enable the testing of anti‐inflammatory and analgesic therapies in a more relevant context than traditional models. The ability to apply mechanical loading while manipulating inflammatory cytokines or testing drug candidates allows for the evaluation of potential therapeutics prior to clinical trials. This approach helps prioritize drug candidates with the highest likelihood of success in clinical trials. Examples of such models include lung‐on‐a‐chip, which simulates respiratory inflammation [73], and gut‐on‐a‐chip, which is used to study inflammatory bowel disease (IBD) [74]. Building on these precedents, disc‐on‐a‐chip systems provide an ideal platform for screening potential therapeutics in a context that replicates the unique biochemical and biomechanical environment of the intervertebral disc. Unlike static cultures, these systems can model degenerative cues, such as hypoxia, low nutrient diffusion, acidic pH, and compressive loading, all of which significantly affect drug responsiveness in disc cells [64]. This level of control enables researchers to examine not only efficacy but also off‐target effects and cellular toxicity under physiologically relevant conditions. For instance, anti‐catabolic drugs targeting IL‐1β or TNF‐α signaling can be tested for their ability to suppress matrix‐degrading enzymes, such as MMPs and ADAMTS, in NP and AF cells [29]. Similarly, senolytic compounds aimed at eliminating senescent disc cells can be evaluated for their impact on reducing SASP factors and preserving matrix homeostasis [153]. These applications are particularly valuable for identifying therapies that modulate inflammation and degeneration in a tissue‐specific manner.

Moreover, the compatibility of disc‐on‐a‐chip systems with patient‐derived cells opens opportunities for personalized medicine, enabling stratification of drug responses based on age, sex, or disease severity [210]. Combined with miniaturized platforms and automated readouts, organ‐on‐a‐chip‐based drug screening can facilitate high‐throughput testing with reduced cost and biological variability, thereby accelerating the translational pipeline for IVD degeneration therapies [67, 75].

### 8.3 Integration With Multi‐Omics Approaches

A major opportunity for advancing organ‐on‐a‐chip models lies in the comprehensive analysis of multi‐omics and computational approaches. Systems biology techniques, such as transcriptomics, proteomics, and metabolomics, can offer deeper insights into the molecular mechanisms underlying IVD degeneration and identify potential key biomarkers for disease progression. Computational models and artificial intelligence (AI) can complement organ‐on‐a‐chip studies by predicting disease trajectories, optimizing experimental conditions, and simulating treatment responses [211].

When applied to disc‐on‐a‐chip platforms, multi‐omics approaches can unravel the complex interactions between mechanical loading, inflammatory signals, and cellular responses in a high‐resolution and data‐rich format. For example, RNA sequencing of cells cultured in organ‐on‐a‐chip devices under degenerative stimuli can reveal key regulators of matrix degradation, senescence, and inflammation. Proteomics can be used to detect secreted factors or ECM components altered during degeneration or after therapeutic intervention, whereas metabolomics can profile shifts in metabolic pathways associated with hypoxia or nutrient deprivation, conditions that closely mimic the avascular disc microenvironment [31]. Single‐cell RNA sequencing (scRNA‐seq) has the potential to map heterogeneous cell populations within the intervertebral disc and their dynamic changes under various experimental conditions. This is particularly relevant for understanding the cellular diversity in degenerated versus healthy disc tissues, including senescent cells, immune infiltrates, and progenitor‐like cell populations [212]. Integrating these data with disc‐on‐a‐chip systems enables a more precise simulation of patient‐specific or stage‐specific disease conditions.

## 9 Toward Clinical Translation

By closely mimicking human disc biology, organ‐on‐a‐chip systems facilitate the translation of preclinical findings into improved clinical outcomes, offering a pathway for more effective and tailored interventions for IVD degeneration. Organ‐on‐a‐chip systems have significantly advanced the study of intervertebral disc degeneration by providing a platform for exploring molecular mechanisms, preclinical drug screening, and personalized medicine applications. Moving toward clinical translation, regulatory approval, validation protocols, and standardization efforts are essential. However, the potential of organ‐on‐a‐chip technology for patient‐specific diagnostic and therapeutic testing offers exciting prospects for precision medicine in the treatment of IVD degeneration. Disc‐on‐a‐chip systems should be evaluated against known mediators of discogenic pain. Key pro‐inflammatory cytokines (IL‐1β, TNF‐α, IL‐6, IL‐17), neurotrophic factors (NGF, BDNF, Substance P, CGRP), and ion channels (TRPV1, ASIC2, Nav1.7/1.8/1.9) represent critical signatures of inflammation‐driven nociception in IVD degeneration [176]. Validation of organ‐on‐a‐chip in vitro models is frequently constrained by the lack of healthy, age‐matched human disc tissues, as surgical specimens are usually deteriorated. Recently, we established a cadaveric dissection protocol to obtain complete lumbar intervertebral discs with retained endplates and minimal age‐related alterations, offering a dependable source of control tissues for comparative research [213]. Despite advancements in organ‐on‐a‐chip technology, particularly in the development of disc‐on‐a‐chip models, several technical, biological, and regulatory challenges should be addressed for their successful implementation in clinical applications.

## 10 Challenges and Future Directions

Although disc‐on‐a‐chip technology has great potential, issues with scalability, material durability, and cell compatibility still exist. Research on long‐term degeneration and inflammation requires the fabrication of devices that can sustain extended mechanical loads without degradation. The lack of standardization in the manufacturing process remains a significant hurdle for organ‐on‐a‐chip and microfluidic‐on‐a‐chip technologies, with no universally accepted protocols, fabrication techniques, or materials in place [76]. This inconsistency affects the reproducibility and reliability of experimental results and delays the scalability and cost‐effectiveness required for commercial production. Variations in device design and material choice between laboratories affect data comparability [77]. A crucial component of IVD degeneration is the pain signaling pathway, which can be better understood by incorporating nociceptive neurons into these models. Furthermore, organ‐on‐a‐chip systems face several technical challenges, particularly in terms of scalability and reproducibility. Standardizing chip fabrication, biomaterial properties, and cell sources remains a significant hurdle in ensuring consistent results across different studies. Variability in biomechanical loading conditions, microfluidic flow, and cellular responses can impact reliability, making it difficult to draw definitive conclusions [214]. Another critical limitation is the difficulty in simulating long‐term degeneration. IVD degeneration is a progressive condition that develops over several years, whereas most organ‐on‐a‐chip models operate over shorter timescales. Maintaining cell viability, extracellular matrix integrity, and chronic inflammatory responses within these systems remains a key research challenge that must be addressed to improve disease modeling. Future research needs to prioritize the establishment of standardized validation criteria to improve the reproducibility as well as physiological relevance of disc‐on‐a‐chip for a preclinical drug screening platform toward clinical translation.

## 11 Conclusion

Disc‐on‐a‐chip models are a major advancement in IVD research, offering a new platform for the controlled study of pain, inflammation, and degeneration. These models address the limitations of traditional in vitro and in vivo systems by accurately mimicking the conditions of the human IVD. Although challenges remain, further progress in tissue engineering and microfabrication will enhance disc‐on‐a‐chip technology, advance the study of inflammation‐driven degeneration, and enable the development of targeted treatments to reduce pain and improve patient outcomes. Organ‐on‐a‐chip models have also transformed preclinical drug screening by supporting the dynamic testing of anti‐inflammatory and analgesic therapies in controlled environments. Additionally, their ability to incorporate patient‐specific cells paves the way for personalized medicine, making treatments more precise and effective than traditional approaches.

The potential of organ‐on‐a‐chip technology to transform IVD degeneration research lies in its ability to capture the complex interactions that drive degeneration and pain, surpassing the limitations of in vitro and animal models. Although challenges such as scalability, long‐term modeling, and regulatory approval remain, advances in multi‐omics integration and computational modeling will enhance their predictive power. With continued refinement, these systems could pave the way for clinically relevant discoveries and accelerate the development of mechanistic disease modeling and therapeutic testing. As organ‐on‐a‐chip platforms evolve, they hold the promise of improving patient outcomes by facilitating more effective and personalized strategies for managing IVD degeneration‐related inflammation and pain. Future disc‐on‐a‐chip platforms must integrate multicellular interactions among the disc cells, sensory neurons, immune cells, and vascular components within the standardized and verified microphysiology systems to improve physiological relevance, reproducibility, and translational applications.

## Author Contributions

I.L.M.I. assisted in article conception, writing, and reviewing. I.Z. assisted in the literature survey and writing the original draft. N.F.Z. assisted in the article writing. A.P. assisted in article reviewing. All the authors provided the final approval.

## Conflicts of Interest

The authors declare no conflicts of interest.

## Data Availability

The authors have nothing to report.

## References

[1] R. J. Gatchel , “The Continuing and Growing Epidemic of Chronic Low Back Pain,” Healthcare 3 (2015): 838–845. 27417800 10.3390/healthcare3030838 PMC4939568

[2] K. M. C. Cheung , J. Karppinen , D. Chan , et al., “Prevalence and Pattern of Lumbar Magnetic Resonance Imaging Changes in a Population Study of One Thousand Forty‐Three Individuals,” Spine 34 (2009): 934–940. 19532001 10.1097/BRS.0b013e3181a01b3f

[3] M. Kanayama , D. Togawa , C. Takahashi , T. Terai , and T. Hashimoto , “Cross‐Sectional Magnetic Resonance Imaging Study of Lumbar Disc Degeneration in 200 Healthy Individuals,” Journal of Neurosurgery: Spine 11 (2009): 501–507. 19929349 10.3171/2009.5.SPINE08675

[4] L. Kalichman , D. H. Kim , L. Li , A. Guermazi , and D. J. Hunter , “Computed Tomography‐Evaluated Features of Spinal Degeneration: Prevalence, Intercorrelation, and Association With Self‐Reported Low Back Pain,” Spine Journal 10 (2010): 200–208. 10.1016/j.spinee.2009.10.018 PMC3686273 20006557

[5] F. Fatoye , T. Gebrye , C. E. Mbada , and U. Useh , “Clinical and Economic Burden of Low Back Pain in Low‐ and Middle‐Income Countries: A Systematic Review,” BMJ Open 13 (2023): e064119. 10.1136/bmjopen-2022-064119 PMC10151982 37185180

[6] M. Molinos , C. R. Almeida , J. Caldeira , C. Cunha , R. M. Gonçalves , and M. A. Barbosa , “Inflammation in Intervertebral Disc Degeneration and Regeneration,” Journal of the Royal Society Interface 12 (2015): 20141191. 25673296 10.1098/rsif.2014.1191 PMC4345483

[7] I. A. F. Stokes and J. C. Iatridis , “Mechanical Conditions That Accelerate Intervertebral Disc Degeneration: Overload Versus Immobilization,” Spine 29 (2004): 2724. 15564921 10.1097/01.brs.0000146049.52152.da PMC7173624

[8] A. S. Morais , M. Mendes , M. A. Cordeiro , et al., “Organ‐on‐a‐Chip: Ubi Sumus? Fundamentals and Design Aspects,” Pharmaceutics 16 (2024): 615. 38794277 10.3390/pharmaceutics16050615 PMC11124787

[9] T. T. Hickman , S. Rathan‐Kumar , and S. H. Peck , “Development, Pathogenesis, and Regeneration of the Intervertebral Disc: Current and Future Insights Spanning Traditional to Omics Methods,” Frontiers in Cell and Developmental Biology 10 (2022): 841831. 35359439 10.3389/fcell.2022.841831 PMC8963184

[10] M. Kmail , R. Razak , and I. L. Mohd Isa , “Engineering Extracellular Matrix‐Based Hydrogels for Intervertebral Disc Regeneration,” Frontiers in Bioengineering and Biotechnology 13 (2025): 1601154. 40375978 10.3389/fbioe.2025.1601154 PMC12078266

[11] D. E. Fournier , P. K. Kiser , J. K. Shoemaker , M. C. Battié , and C. A. Séguin , “Vascularization of the Human Intervertebral Disc: A Scoping Review,” JOR Spine 3 (2020): e1123. 33392458 10.1002/jsp2.1123 PMC7770199

[12] B. Alkhatib , G. I. Ban , S. Williams , and R. Serra , “IVD Development: Nucleus Pulposus Development and Sclerotome Specification,” Current Molecular Biology Reports 4 (2018): 132–141. 30505649 10.1007/s40610-018-0100-3 PMC6261384

[13] Y. Dou , X. Sun , X. Ma , X. Zhao , and Q. Yang , “Intervertebral Disk Degeneration: The Microenvironment and Tissue Engineering Strategies,” Frontiers in Bioengineering and Biotechnology 9 (2021): 592118. 34354983 10.3389/fbioe.2021.592118 PMC8329559

[14] I. L. Mohd Isa , S. L. Teoh , N. H. Mohd Nor , and S. A. Mokhtar , “Discogenic Low Back Pain: Anatomy, Pathophysiology and Treatments of Intervertebral Disc Degeneration,” International Journal of Molecular Sciences 24 (2023): 208. 10.3390/ijms24010208 PMC9820240 36613651

[15] M. P. Steinmetz and E. C. Benzel , eds., Benzel’s Spine Surgery: Techniques, Complication Avoidance, and Management, 4th ed. (Elsevier, 2016).

[16] W. M. Erwin , K. Ashman , P. O’Donnel , and R. D. Inman , “Nucleus Pulposus Notochord Cells Secrete Connective Tissue Growth Factor and Up‐Regulate Proteoglycan Expression by Intervertebral Disc Chondrocytes,” Arthritis & Rheumatism 54 (2006): 3859–3867. 17136753 10.1002/art.22258

[17] F. Bach , S. Libregts , L. Creemers , et al., “Notochordal‐Cell Derived Extracellular Vesicles Exert Regenerative Effects on Canine and Human Nucleus Pulposus Cells,” Oncotarget 8 (2017): 88845–88856. 29179481 10.18632/oncotarget.21483 PMC5687651

[18] M. R. McCann and C. A. Séguin , “Notochord Cells in Intervertebral Disc Development and Degeneration,” Journal of Developmental Biology 4 (2016): 3. 27252900 10.3390/jdb4010003 PMC4885739

[19] G. G. H. Van Den Akker , D. A. M. Surtel , A. Cremers , et al., “Novel Immortal Cell Lines Support Cellular Heterogeneity in the Human Annulus Fibrosus,” PLoS One 11 (2016): e0144497. 26794306 10.1371/journal.pone.0144497 PMC4721917

[20] J. A. Waxenbaum , V. Reddy , and B. Futterman , “Anatomy, Back, Intervertebral Discs,” in StatPearls [Internet] (StatPearls Publishing, 2025). 29262063

[21] J. Tavakoli , “Tissue Engineering of the Intervertebral Disc’s Annulus Fibrosus: A Scaffold‐Based Review Study,” Tissue Engineering and Regenerative Medicine 14 (2017): 81–91. 30603465 10.1007/s13770-017-0024-7 PMC6171584

[22] S. I. Sinopoli , M. C. Whittal , N. Chow , and D. E. Gregory , “Mechanical Age‐Related Differences in the Human Cadaveric Annulus Fibrosus,” Journal of the Mechanical Behavior of Biomedical Materials 168 (2025): 107015. 40267691 10.1016/j.jmbbm.2025.107015

[23] K. B. Crump , A. Alminnawi , P. Bermudez‐Lekerika , et al., “Cartilaginous Endplates: A Comprehensive Review on a Neglected Structure in Intervertebral Disc Research,” JOR Spine 6 (2023): e1294. 38156054 10.1002/jsp2.1294 PMC10751983

[24] S. M. Moon , J. H. Yoder , A. C. Wright , L. J. Smith , E. J. Vresilovic , and D. M. Elliott , “Evaluation of Intervertebral Disc Cartilaginous Endplate Structure Using Magnetic Resonance Imaging,” European Spine Journal 22 (2013): 1820–1828. 23674162 10.1007/s00586-013-2798-1 PMC3731490

[25] H. Yang , X. Chen , J. Chen , et al., “The Pathogenesis and Targeted Therapies of Intervertebral Disc Degeneration Induced by Cartilage Endplate Inflammation,” Frontiers in Cell and Developmental Biology 12 (2024): 1492870. 39687521 10.3389/fcell.2024.1492870 PMC11647014

[26] J. F. DeLucca , D. H. Cortes , N. T. Jacobs , E. J. Vresilovic , R. L. Duncan , and D. M. Elliott , “Human Cartilage Endplate Permeability Varies With Degeneration and Intervertebral Disc Site,” Journal of Biomechanics 49 (2016): 550–557. 26874969 10.1016/j.jbiomech.2016.01.007 PMC4779374

[27] K. Lakstins , L. Arnold , G. Gunsch , et al., “Characterization of the Human Intervertebral Disc Cartilage Endplate at the Molecular, Cell, and Tissue Levels,” Journal of Orthopaedic Research 39 (2021): 1898–1907. 32915471 10.1002/jor.24854

[28] K. Wuertz and L. Haglund , “Inflammatory Mediators in Intervertebral Disk Degeneration and Discogenic Pain,” Global Spine Journal 3 (2013): 175–184. 24436868 10.1055/s-0033-1347299 PMC3854585

[29] Z. I. Johnson , Z. R. Schoepflin , H. Choi , I. M. Shapiro , and M. V. Risbud , “Disc in Flames: Roles of TNF‐α and IL‐1β in Intervertebral Disc Degeneration,” European Cells and Materials 30 (2015): 104–117. 26388614 10.22203/ecm.v030a08 PMC4751407

[30] Y. Wang , M. Che , J. Xin , Z. Zheng , J. Li , and S. Zhang , “The Role of IL‐1β and TNF‐α in Intervertebral Disc Degeneration,” Biomedicine & Pharmacotherapy 131 (2020): 110660. 32853910 10.1016/j.biopha.2020.110660

[31] M. V. Risbud and I. M. Shapiro , “Role of Cytokines in Intervertebral Disc Degeneration: Pain and Disc‐Content,” Nature Reviews Rheumatology 10 (2014): 44. 24166242 10.1038/nrrheum.2013.160 PMC4151534

[32] A. Mukherjee and B. Das , “The Role of Inflammatory Mediators and Matrix Metalloproteinases (MMPs) in the Progression of Osteoarthritis,” Biomaterials and Biosystems 13 (2024): 100090. 38440290 10.1016/j.bbiosy.2024.100090 PMC10910010

[33] C. W. Frevert , J. Felgenhauer , M. Wygrecka , M. V. Nastase , and L. Schaefer , “Danger‐Associated Molecular Patterns Derived From the Extracellular Matrix Provide Temporal Control of Innate Immunity,” Journal of Histochemistry & Cytochemistry 66 (2018): 213. 29290139 10.1369/0022155417740880 PMC5958376

[34] C. Lambert , J. Zappia , C. Sanchez , A. Florin , J. E. Dubuc , and Y. Henrotin , “The Damage‐Associated Molecular Patterns (DAMPs) as Potential Targets to Treat Osteoarthritis: Perspectives From a Review of the Literature,” Frontiers of Medicine 7 (2020): 607186. 10.3389/fmed.2020.607186 PMC7847938 33537330

[35] P. Silwal , A. M. Nguyen‐Thai , H. A. Mohammad , et al., “Cellular Senescence in Intervertebral Disc Aging and Degeneration: Molecular Mechanisms and Potential Therapeutic Opportunities,” Biomolecules 13 (2023): 686. 37189433 10.3390/biom13040686 PMC10135543

[36] A. Latremoliere and C. J. Woolf , “Central Sensitization: A Generator of Pain Hypersensitivity by Central Neural Plasticity,” Journal of Pain 10 (2009): 895–926. 19712899 10.1016/j.jpain.2009.06.012 PMC2750819

[37] F. A. Russell , R. King , S. J. Smillie , X. Kodji , and S. D. Brain , “Calcitonin Gene‐Related Peptide: Physiology and Pathophysiology,” Physiological Reviews 94 (2014): 1099–1142. 25287861 10.1152/physrev.00034.2013 PMC4187032

[38] S. Saleem , H. M. Aslam , M. A. K. Rehmani , A. Raees , A. A. Alvi , and J. Ashraf , “Lumbar Disc Degenerative Disease: Disc Degeneration Symptoms and Magnetic Resonance Image Findings,” Asian Spine Journal 7 (2013): 322–334. 24353850 10.4184/asj.2013.7.4.322 PMC3863659

[39] I. L. Mohd Isa , S. A. Mokhtar , S. A. Abbah , M. B. Fauzi , A. Devitt , and A. Pandit , “Intervertebral Disc Degeneration: Biomaterials and Tissue Engineering Strategies Toward Precision Medicine,” Advanced Healthcare Materials 11 (2022): e2102530. 35373924 10.1002/adhm.202102530 PMC11469247

[40] E. D. Rivera Tapia , J. R. Meakin , and T. P. Holsgrove , “In‐Vitro Models of Disc Degeneration – A Review of Methods and Clinical Relevance,” Journal of Biomechanics 142 (2022): 111260. 36027637 10.1016/j.jbiomech.2022.111260

[41] H. S. An and K. Masuda , “Relevance of In Vitro and In Vivo Models for Intervertebral Disc Degeneration,” Journal of Bone and Joint Surgery 88 (2006): 88–94. 10.2106/JBJS.E.01272 16595451

[42] A. Mainardi , E. Cambria , P. Occhetta , et al., “Intervertebral Disc‐on‐a‐Chip as Advanced In Vitro Model for Mechanobiology Research and Drug Testing: A Review and Perspective,” Frontiers in Bioengineering and Biotechnology 9 (2021): 826867. 35155416 10.3389/fbioe.2021.826867 PMC8832503

[43] S. C. W. Chan , A. Bürki , H. M. Bonél , L. M. Benneker , and B. Gantenbein‐Ritter , “Papain‐Induced In Vitro Disc Degeneration Model for the Study of Injectable Nucleus Pulposus Therapy,” Spine Journal 13 (2013): 273–283. 10.1016/j.spinee.2012.12.007 23353003

[44] R. Bostelmann , H. J. Steiger , and J. F. Cornelius , “Effect of Annular Defects on Intradiscal Pressures in the Lumbar Spine: An In Vitro Biomechanical Study of Diskectomy and Annular Repair,” Journal of Neurological Surgery Part A: Central European Neurosurgery 78 (2017): 46–52. 27123747 10.1055/s-0035-1570344

[45] C. Techens , M. Palanca , P. E. Éltes , Á Lazáry , and L. Cristofolini , “Testing the Impact of Discoplasty on the Biomechanics of the Intervertebral Disc With Simulated Degeneration: An In Vitro Study,” Medical Engineering & Physics 84 (2020): 51–59. 32977922 10.1016/j.medengphy.2020.07.024

[46] J. W. Liu , K. H. Lin , C. Weber , et al., “An In Vitro Organ Culture Model of the Murine Intervertebral Disc,” *JoVE* (Journal of Visualized Experiments) 11 (2017): 55437. 10.3791/55437 PMC5495460 28448052

[47] A. Slanzi , G. Iannoto , B. Rossi , E. Zenaro , and G. Constantin , “In Vitro Models of Neurodegenerative Diseases,” Frontiers in Cell and Developmental Biology 8 (2020): 328. 32528949 10.3389/fcell.2020.00328 PMC7247860

[48] S. M. Romereim , C. A. Johnston , A. L. Redwine , and R. A. Wachs , “Development of an In Vitro Intervertebral Disc Innervation Model to Screen Neuroinhibitory Biomaterials,” Journal of Orthopaedic Research 38 (2020): 1016–1026. 31825104 10.1002/jor.24557 PMC7244214

[49] N. Rashidi , N. Dowell , D. Covill , J. Shepperd , and M. Santin , “Biomimetic Hydrogels for In Vitro Modelling of Nucleus Pulposus Degeneration: Effects of Extracellular Matrix Compositional Change on Physicochemical Properties and Cell Phenotype,” Journal of Functional Biomaterials 16 (2025): 253. 40710467 10.3390/jfb16070253 PMC12295825

[50] S. Yan , Y. Lu , C. An , et al., “Biomechanical Research Using Advanced Micro‐Nano Devices: In‐Vitro Cell Characterization Focus,” Journal of Advanced Research 76 (2025): 615. 39701378 10.1016/j.jare.2024.12.024 PMC12793746

[51] W. Jiang , J. D. Glaeser , G. Kaneda , et al., “Intervertebral Disc Human Nucleus Pulposus Cells Associated With Back Pain Trigger Neurite Outgrowth In Vitro and Pain Behaviors in Rats,” Science Translational Medicine 15 (2023): eadg7020. 38055799 10.1126/scitranslmed.adg7020 PMC12083434

[52] M. H. Hwang , H. G. Son , J. Kim , and H. Choi , “In Vitro Model of Distinct Catabolic and Inflammatory Response Patterns of Endothelial Cells to Intervertebral Disc Cell Degeneration,” Scientific Reports 10 (2020): 20596. 33244116 10.1038/s41598-020-77785-6 PMC7691345

[53] D. Khosrowshahi , L. Lagae , and J. Bolander , “Decoding Pain: Next‐Generation In Vitro Systems for Mechanistic Insights and Drug Discovery,” FASEB Journal 39 (2025): e70914. 40814286 10.1096/fj.202501025RR PMC12355202

[54] E. Salzer , T. C. Schmitz , V. H. M. Mouser , et al., “Ex Vivo Intervertebral Disc Cultures: Degeneration‐Induction Methods and Their Implications for Clinical Translation,” European Cells and Materials 45 (2023): 88–112. 36989118 10.22203/eCM.v045a07

[55] M. Peroglio , L. S. Douma , T. S. Caprez , et al., “Intervertebral Disc Response to Stem Cell Treatment Is Conditioned by Disc State and Cell Carrier: An *Ex Vivo* Study,” Journal of Orthopaedic Translation 9 (2017): 43–51. 29662798 10.1016/j.jot.2017.03.003 PMC5822953

[56] Z. Li , P. Lezuo , G. Pattappa , et al., “Development of an Ex Vivo Cavity Model to Study Repair Strategies in Loaded Intervertebral Discs,” European Spine Journal 25 (2016): 2898–2908. 27037921 10.1007/s00586-016-4542-0

[57] E. E. McDonnell and C. T. Buckley , “Investigating the Physiological Relevance of Ex Vivo Disc Organ Culture Nutrient Microenvironments Using in Silico Modeling and Experimental Validation,” JOR Spine 4 (2021): e1141. 34337330 10.1002/jsp2.1141 PMC8313156

[58] Z. Li , Y. Gehlen , F. Heizmann , et al., “Preclinical Ex‐Vivo Testing of Anti‐Inflammatory Drugs in a Bovine Intervertebral Degenerative Disc Model,” Frontiers in Bioengineering and Biotechnology 8 (2020): 583. 32587853 10.3389/fbioe.2020.00583 PMC7298127

[59] S. N. Tang , A. F. Bonilla , N. O. Chahine , et al., “Controversies in Spine Research: Organ Culture Versus In Vivo Models for Studies of the Intervertebral Disc,” JOR Spine 5 (2022): e1235. 36601369 10.1002/jsp2.1235 PMC9799089

[60] D. L. Poletto , J. D. Crowley , O. Tanglay , W. R. Walsh , and M. H. Pelletier , “Preclinical In Vivo Animal Models of Intervertebral Disc Degeneration. Part 1: A Systematic Review,” JOR Spine 6 (2023): e1234. 36994459 10.1002/jsp2.1234 PMC10041387

[61] A. Lai , D. Iliff , K. Zaheer , et al., “Spinal Cord Sensitization and Spinal Inflammation From an In Vivo Rat Endplate Injury Associated With Painful Intervertebral Disc Degeneration,” International Journal of Molecular Sciences 24 (2023): 3425. 36834838 10.3390/ijms24043425 PMC9964286

[62] L. E. Lisiewski , H. E. Jacobsen , D. C. M. Viola , H. M. Kenawy , D. N. Kiridly , and N. O. Chahine , “Intradiscal Inflammatory Stimulation Induces Spinal Pain Behavior and Intervertebral Disc Degeneration In Vivo,” FASEB Journal 38 (2024): e23364. 38091247 10.1096/fj.202300227R PMC10795732

[63] C. Olaizola‐Rodrigo , C. Bayona , S. Oliván , and R. Monge , “A Review of Organ‐on‐Chip Fabrication Methods: From Early Developments to Overcoming Inert Barriers,” iScience 28 (2025): 113992. 41377670 10.1016/j.isci.2025.113992 PMC12689227

[64] J. Dai , Y. Xing , L. Xiao , et al., “Microfluidic Disc‐on‐a‐Chip Device for Mouse Intervertebral Disc—Pitching a Next‐Generation Research Platform to Study Disc Degeneration,” ACS Biomaterials Science & Engineering 5 (2019): 2041–2051. 31763444 10.1021/acsbiomaterials.8b01522 PMC6874371

[65] J. P. McKinley , A. R. Montes , M. N. Wang , et al., “Design of a Flexing Organ‐Chip to Model In Situ Loading of the Intervertebral Disc,” Biomicrofluidics 16 (2022): 054111. 36330201 10.1063/5.0103141 PMC9625834

[66] W. Xie , Y. Xing , L. Xiao , et al., “Intervertebral Disc‐on‐a‐Chip^MF^: A New Model for Mouse Disc Culture via Integrating Mechanical Loading and Dynamic Media Flow,” Advanced Materials Technologies 8 (2023): 2300606. 39130370 10.1002/admt.202300606 PMC11315454

[67] B. Zhang , A. Korolj , B. F. L. Lai , and M. Radisic , “Advances in Organ‐on‐a‐Chip Engineering,” Nature Reviews Materials 3 (2018): 257–278.

[68] J. H. Shin , M. H. Hwang , S. M. Back , et al., “Electrical Impulse Effects on Degenerative Human Annulus Fibrosus Model to Reduce Disc Pain Using Micro‐Electrical Impulse‐on‐a‐Chip,” Scientific Reports 9 (2019): 5827. 30967598 10.1038/s41598-019-42320-9 PMC6456732

[69] H. G. Son , M. H. Hwang , S. Lee , et al., “Intervertebral Disc Organ‐on‐a‐Chip: An Innovative Model to Study Monocyte Extravasation During Nucleus Pulposus Degeneration,” Lab on a Chip 23 (2023): 2819–2828. 37212601 10.1039/d3lc00032j

[70] M. H. Hwang , D. H. Cho , S. M. Baek , et al., “Spine‐on‐a‐Chip: Human Annulus Fibrosus Degeneration Model for Simulating the Severity of Intervertebral Disc Degeneration,” Biomicrofluidics 11 (2017): 064107. 29250209 10.1063/1.5005010 PMC5718916

[71] V. Kumar , D. Kingsley , S. M. Perikamana , P. Mogha , C. R. Goodwin , and S. Varghese , “Self‐Assembled Innervated Vasculature‐on‐a‐Chip to Study Nociception,” Biofabrication 15 (2023): 035008. 10.1088/1758-5090/acc904 PMC10152403 36996841

[72] S. Kim , H. Lee , M. Chung , and N. L. Jeon , “Engineering of Functional, Perfusable 3D Microvascular Networks on a Chip,” Lab on a Chip 13 (2013): 1489–1500. 23440068 10.1039/c3lc41320a

[73] L. van Os , J. Yeoh , G. Witz , et al., “Immune Cell Extravasation in an Organ‐on‐Chip to Model Lung Inflammation,” European Journal of Pharmaceutical Sciences 187 (2023): 106485. 37270149 10.1016/j.ejps.2023.106485 PMC10234692

[74] S. Taavitsainen , K. Juuti‐Uusitalo , K. Kurppa , K. Lindfors , P. Kallio , and M. Kellomäki , “Gut‐on‐Chip Devices as Intestinal Inflammation Models and Their Future for Studying Multifactorial Diseases,” Frontiers in Lab on a Chip Technologies 2 (2023): 1337945.

[75] L. A. Low and D. A. Tagle , “Organs‐On‐Chips: Progress, Challenges, and Future Directions,” Experimental Biology and Medicine 242 (2017): 1573–1578. 28343437 10.1177/1535370217700523 PMC5661765

[76] C. Ma , Y. Peng , H. Li , and W. Chen , “Organ‐on‐a‐Chip: A New Paradigm for Drug Development,” Trends in Pharmacological Sciences 42 (2021): 119–133. 33341248 10.1016/j.tips.2020.11.009 PMC7990030

[77] S. K. Srivastava , G. W. Foo , N. Aggarwal , and M. W. Chang , “Organ‐on‐Chip Technology: Opportunities and Challenges,” Biotechnology Notes 5 (2024): 8–12. 39416695 10.1016/j.biotno.2024.01.001 PMC11446384

[78] M. H. Hwang , Y. J. Kang , H. G. Son , H. Cho , and H. Choi , “Engineered Human Intervertebral Disc Model Inducing Degenerative Microglial Proinflammation,” International Journal of Molecular Sciences 23 (2022): 12216. 36293070 10.3390/ijms232012216 PMC9603448

[79] J. Du , J. J. Pfannkuche , G. Lang , et al., “Proinflammatory Intervertebral Disc Cell and Organ Culture Models Induced by Tumor Necrosis Factor Alpha,” JOR Spine 3 (2020): e1104. 33015577 10.1002/jsp2.1104 PMC7524256

[80] Y. Gong , J. Qiu , T. Jiang , et al., “Maltol Ameliorates Intervertebral Disc Degeneration Through Inhibiting PI3K/AKT/NF‐κB Pathway and Regulating NLRP3 Inflammasome‐Mediated Pyroptosis,” Inflammopharmacology 31 (2023): 369–384. 36401729 10.1007/s10787-022-01098-5 PMC9957850

[81] A. Larrañaga , I. L. M. Isa , V. Patil , et al., “Antioxidant Functionalized Polymer Capsules to Prevent Oxidative Stress,” Acta Biomaterialia 67 (2018): 21–31. 29258803 10.1016/j.actbio.2017.12.014

[82] I. L. M. Isa , A. Srivastava , D. Tiernan , et al., “Hyaluronic Acid Based Hydrogels Attenuate Inflammatory Receptors and Neurotrophins in Interleukin‐1β Induced Inflammation Model of Nucleus Pulposus Cells,” Biomacromolecules 16 (2015): 1714–1725. 25871410 10.1021/acs.biomac.5b00168

[83] E. Salzer , V. H. M. Mouser , M. A. Tryfonidou , and K. Ito , “A Bovine Nucleus Pulposus Explant Culture Model,” Journal of Orthopaedic Research 40 (2022): 2089–2102. 34812520 10.1002/jor.25226 PMC9542046

[84] R. Gawri , F. Mwale , J. Ouellet , et al., “Development of an Organ Culture System for Long‐Term Survival of the Intact Human Intervertebral Disc,” Spine 36 (2011): 1835–1842. 21270705 10.1097/BRS.0b013e3181f81314

[85] M. N. Barcellona , T. N. Néill , F. O’Brien , J. E. Dixon , C. Curtin , and C. Buckley , “Modulation of Inflammation and Regeneration in the Intervertebral Disc Using Enhanced Cell‐Penetrating Peptides for MicroRNA Delivery,” Advanced NanoBiomed Research 4 (2024): 2300112.

[86] D. M. Elliott , C. S. Yerramalli , J. C. Beckstein , J. I. Boxberger , W. Johannessen , and E. J. Vresilovic , “The Effect of Relative Needle Diameter in Puncture and Sham Injection Animal Models of Degeneration,” Spine 33 (2008): 588. 18344851 10.1097/BRS.0b013e318166e0a2

[87] M. Inoue , I. L. M. Isa , S. Orita , et al., “An Injectable Hyaluronic Acid Hydrogel Promotes Intervertebral Disc Repair in a Rabbit Model,” Spine 46 (2021): E810. 34228691 10.1097/BRS.0000000000003921

[88] R. Mohd Razak , N. A. A. Harizal , M. A. Z. Azman , et al., “Deciphering the Effect of Hyaluronic Acid/Collagen Hydrogel for Pain Relief and Tissue Hydration in a Rat Model of Intervertebral Disc Degeneration,” Polymers 16 (2024): 2574. 39339037 10.3390/polym16182574 PMC11435062

[89] I. L. Mohd Isa , S. A. Abbah , M. Kilcoyne , et al., “Implantation of Hyaluronic Acid Hydrogel Prevents the Pain Phenotype in a Rat Model of Intervertebral Disc Injury,” Science Advances 4 (2018): eaaq0597. 29632893 10.1126/sciadv.aaq0597 PMC5884685

[90] A. Friedmann , A. Baertel , C. Schmitt , et al., “Intervertebral Disc Regeneration Injection of a Cell‐Loaded Collagen Hydrogel in a Sheep Model,” International Journal of Molecular Sciences 22 (2021): 4248. 33921913 10.3390/ijms22084248 PMC8072963

[91] F. J. Lyu , H. Cui , H. Pan , et al., “Painful Intervertebral Disc Degeneration and Inflammation: From Laboratory Evidence to Clinical Interventions,” Bone Research 9 (2021): 7. 33514693 10.1038/s41413-020-00125-x PMC7846842

[92] D. Lazaro‐Pacheco , M. Mohseni , S. Rudd , J. Cooper‐White , and T. P. Holsgrove , “The Role of Biomechanical Factors in Models of Intervertebral Disc Degeneration Across Multiple Length Scales,” APL Bioengineering 7 (2023): 021501. 37180733 10.1063/5.0137698 PMC10168717

[93] F. Hendijani , “Explant Culture: An Advantageous Method for Isolation of Mesenchymal Stem Cells From Human Tissues,” Cell Proliferation 50 (2017): e12334. 28144997 10.1111/cpr.12334 PMC6529062

[94] F. C. Walker and I. Derré , “Contributions of Diverse Models of the Female Reproductive Tract to the Study of *Chlamydia trachomatis*‐Host Interactions,” Current Opinion in Microbiology 77 (2024): 102416. 38103413 10.1016/j.mib.2023.102416 PMC10922760

[95] J. T. Stannard , K. Edamura , A. M. Stoker , et al., “Development of a Whole Organ Culture Model for Intervertebral Disc Disease,” Journal of Orthopaedic Translation 5 (2016): 1–8. 30035069 10.1016/j.jot.2015.08.002 PMC5987001

[96] J. J. Pfannkuche , W. Guo , S. Cui , et al., “Intervertebral Disc Organ Culture for the Investigation of Disc Pathology and Regeneration – Benefits, Limitations, and Future Directions of Bioreactors,” Connective Tissue Research 61 (2020): 304–321. 31556329 10.1080/03008207.2019.1665652

[97] Z. Li , G. Lang , X. Chen , et al., “Polyurethane Scaffold With *In Situ* Swelling Capacity for Nucleus Pulposus Replacement,” Biomaterials 84 (2016): 196–209. 26828684 10.1016/j.biomaterials.2016.01.040

[98] S. X. Gu , X. Li , J. L. Hamilton , et al., “MicroRNA‐146a Reduces IL‐1 Dependent Inflammatory Responses in the Intervertebral Disc,” Gene 555 (2015): 80–87. 25311550 10.1016/j.gene.2014.10.024 PMC4495656

[99] C. Leite Pereira , G. Quelhas Teixeira , J. Rita Ferreira , et al., “Stromal Cell Derived Factor‐1‐Mediated Migration of Mesenchymal Stem Cells Enhances Collagen Type II Expression in Intervertebral Disc,” Tissue Engineering Part A 24 (2018): 1818–1830. 10.1089/ten.TEA.2018.0131 29916307

[100] D. Purmessur , B. A. Walter , P. J. Roughley , D. M. Laudier , A. C. Hecht , and J. C. Iatridis , “A Role for TNFα in Intervertebral Disc Degeneration: A Non‐Recoverable Catabolic Shift,” Biochemical and Biophysical Research Communications 433 (2013): 151–156. 23438440 10.1016/j.bbrc.2013.02.034 PMC3640343

[101] B. A. Walter , M. Likhitpanichkul , S. Illien‐Junger , P. J. Roughley , A. C. Hecht , and J. C. Iatridis , “TNFα Transport Induced by Dynamic Loading Alters Biomechanics of Intact Intervertebral Discs,” PLoS One 10 (2015): e0118358. 25734788 10.1371/journal.pone.0118358 PMC4348425

[102] D. A. Frauchiger , S. C. W. Chan , L. M. Benneker , and B. Gantenbein , “Intervertebral Disc Damage Models in Organ Culture: A Comparison of Annulus Fibrosus Cross‐Incision Versus Punch Model Under Complex Loading,” European Spine Journal 27 (2018): 1785–1797. 29789921 10.1007/s00586-018-5638-5

[103] R. Gawri , J. Moir , J. Ouellet , et al., “Physiological Loading Can Restore the Proteoglycan Content in a Model of Early IVD Degeneration,” PLoS One 9 (2014): e101233. 24992586 10.1371/journal.pone.0101233 PMC4081577

[104] G. Paesold , A. G. Nerlich , and N. Boos , “Biological Treatment Strategies for Disc Degeneration: Potentials and Shortcomings,” European Spine Journal 16 (2007): 447–468. 16983559 10.1007/s00586-006-0220-y PMC2229827

[105] S. Caprez , U. Menzel , Z. Li , S. Grad , M. Alini , and M. Peroglio , “Isolation of High‐Quality RNA From Intervertebral Disc Tissue via Pronase Predigestion and Tissue Pulverization,” JOR Spine 1 (2018): e1017. 31463444 10.1002/jsp2.1017 PMC6686795

[106] E. C. Bryda , “The Mighty Mouse: The Impact of Rodents on Advances in Biomedical Research,” Missouri Medicine 110 (2013): 207–211. 23829104 PMC3987984

[107] J. Y. Hong , H. Kim , W. J. Jeon , et al., “Animal Models of Intervertebral Disc Diseases: Advantages, Limitations, and Future Directions,” Neurology International 16 (2024): 1788–1818. 39728755 10.3390/neurolint16060129 PMC11678881

[108] Y. Zhang , C. Xiong , M. Kudelko , et al., “Early Onset of Disc Degeneration in SM/J Mice Is Associated With Changes in Ion Transport Systems and Fibrotic Events,” Matrix Biology 70 (2018): 123–139. 29649547 10.1016/j.matbio.2018.03.024

[109] T. Yurube , T. Takada , T. Suzuki , et al., “Rat Tail Static Compression Model Mimics Extracellular Matrix Metabolic Imbalances of Matrix Metalloproteinases, Aggrecanases, and Tissue Inhibitors of Metalloproteinases in Intervertebral Disc Degeneration,” Arthritis Research & Therapy 14 (2012): R51. 22394620 10.1186/ar3764 PMC3446417

[110] Y. Ji , P. Zhu , L. Zhang , and H. Yang , “A Novel Rat Tail Disc Degeneration Model Induced by Static Bending and Compression,” Animal Models and Experimental Medicine 4 (2021): 261–267. 34557652 10.1002/ame2.12178 PMC8446698

[111] K. Sao and M. V. Risbud , “Proteoglycan Dysfunction: A Common Link Between Intervertebral Disc Degeneration and Skeletal Dysplasia,” Neurospine 21 (2024): 162–178. 38569642 10.14245/ns.2347342.671 PMC10992626

[112] A. M. Malfait , J. Ritchie , A. S. Gil , et al., “ADAMTS‐5 Deficient Mice Do Not Develop Mechanical Allodynia Associated With Osteoarthritis Following Medial Meniscal Destabilization,” Osteoarthritis and Cartilage 18 (2010): 572–580. 20036347 10.1016/j.joca.2009.11.013

[113] H. Choi , S. Tessier , E. S. Silagi , et al., “A Novel Mouse Model of Intervertebral Disc Degeneration Shows Altered Cell Fate and Matrix Homeostasis,” Matrix Biology 70 (2018): 102–122. 29605718 10.1016/j.matbio.2018.03.019 PMC6081256

[114] A. K. Kiani , D. Pheby , G. Henehan , et al., “Ethical Considerations Regarding Animal Experimentation,” Journal of Preventive Medicine and Hygiene 63 (2022): E255–E266. 36479489 10.15167/2421-4248/jpmh2022.63.2S3.2768 PMC9710398

[115] I. Ribitsch , P. M. Baptista , A. Lange‐Consiglio , et al., “Large Animal Models in Regenerative Medicine and Tissue Engineering: To Do or Not to Do,” Frontiers in Bioengineering and Biotechnology 8 (2020): 972. 32903631 10.3389/fbioe.2020.00972 PMC7438731

[116] C. He , F. Lu , Y. Liu , Y. Lei , X. Wang , and N. Tang , “Emergent Trends in Organ‐on‐a‐Chip Applications for Investigating Metastasis Within Tumor Microenvironment: A Comprehensive Bibliometric Analysis,” Heliyon 10 (2023): e23504. 38187238 10.1016/j.heliyon.2023.e23504 PMC10770560

[117] D. Zhu , A. Nilghaz , Z. Tong , et al., “Pain‐on‐a‐Chip: A Microfluidic Device for Neuron Differentiation and Functional Discrimination in Animal Models of Chronic Pain,” Biosensors and Bioelectronics 279 (2025): 117401. 40139049 10.1016/j.bios.2025.117401

[118] K. Watanabe , K. Larsson , B. Rydevik , S. I. Konno , C. Nordborg , and K. Olmarker , “Increase of Sodium Channels (Nav 1.8 and Nav 1.9) in Rat Dorsal Root Ganglion Neurons Exposed to Autologous Nucleus Pulposus,” Open Orthopaedics Journal 8 (2014): 69–73. 24843387 10.2174/1874325001408010069 PMC4023406

[119] M. Daigneault , J. A. Preston , H. M. Marriott , M. K. B. Whyte , and D. H. Dockrell , “The Identification of Markers of Macrophage Differentiation in PMA‐Stimulated THP‐1 Cells and Monocyte‐Derived Macrophages,” PLoS One 5 (2010): e8668. 20084270 10.1371/journal.pone.0008668 PMC2800192

[120] G. M. Whitesides , “The Origins and the Future of Microfluidics,” Nature 442 (2006): 368–373. 16871203 10.1038/nature05058

[121] D. Qin , Y. Xia , and G. M. Whitesides , “Soft Lithography for Micro‐ and Nanoscale Patterning,” Nature Protocols 5(2010): 491–502. 20203666 10.1038/nprot.2009.234

[122] N. Bhattacharjee , A. Urrios , S. Kang , and A. Folch , “The Upcoming 3D‐Printing Revolution in Microfluidics,” Lab on a Chip 16 (2016): 1720–1742. 27101171 10.1039/c6lc00163g PMC4862901

[123] H. Becker and C. Gärtner , “Polymer Microfabrication Technologies for Microfluidic Systems,” Analytical and Bioanalytical Chemistry 390 (2008): 89–111. 17989961 10.1007/s00216-007-1692-2

[124] M. D. Poskus , T. Wang , Y. Deng , S. Borcherding , J. Atkinson , and I. K. Zervantonakis , “Fabrication of 3D‐printed Molds for Polydimethylsiloxane‐Based Microfluidic Devices Using a Liquid Crystal Display‐Based Vat Photopolymerization Process: Printing Quality, Drug Response and 3D Invasion Cell Culture Assays,” Microsystems & Nanoengineering 9 (2023): 140. 37954040 10.1038/s41378-023-00607-y PMC10632127

[125] I. Miranda , A. Souza , P. Sousa , et al., “Properties and Applications of PDMS for Biomedical Engineering: A Review,” Journal of Functional Biomaterials 13 (2022): 2. 10.3390/jfb13010002 PMC8788510 35076525

[126] S. Halldorsson , E. Lucumi , R. Gómez‐Sjöberg , and R. M. T. Fleming , “Advantages and Challenges of Microfluidic Cell Culture in Polydimethylsiloxane Devices,” Biosensors and Bioelectronics 63 (2015): 218–231. 25105943 10.1016/j.bios.2014.07.029

[127] J. B. Nielsen , R. L. Hanson , H. M. Almughamsi , C. Pang , T. R. Fish , and A. T. Woolley , “Microfluidics: Innovations in Materials and Their Fabrication and Functionalization,” Analytical Chemistry 92 (2019): 150–168. 31721565 10.1021/acs.analchem.9b04986 PMC7034066

[128] N. Convery and N. Gadegaard , “30 Years of Microfluidics,” Micro and Nano Engineering 2 (2019): 76–91.

[129] S. B. Campbell , Q. Wu , J. Yazbeck , C. Liu , S. Okhovatian , and M. Radisic , “Beyond Polydimethylsiloxane: Alternative Materials for Fabrication of Organ‐on‐a‐Chip Devices and Microphysiological Systems,” ACS Biomaterials Science & Engineering 7 (2021): 2880–2899. 34275293 10.1021/acsbiomaterials.0c00640

[130] S. Nagarajan , H. Belaid , S. Radhakrishnan , et al., “Sacrificial Mold‐Assisted 3D Printing of Stable Biocompatible Gelatin Scaffolds,” Bioprinting 22 (2021): e00140.

[131] S. Liu , T. Wang , S. Li , and X. Wang , “Application Status of Sacrificial Biomaterials in 3D Bioprinting,” Polymers 14 (2022): 2182. 35683853 10.3390/polym14112182 PMC9182955

[132] H. Budharaju , D. Sundaramurthi , and S. Sethuraman , “Embedded 3D Bioprinting – An Emerging Strategy to Fabricate Biomimetic & Large Vascularized Tissue Constructs,” Bioactive Materials 32 (2024): 356–384. 37920828 10.1016/j.bioactmat.2023.10.012 PMC10618244

[133] C. Tomasina , T. Bodet , C. Mota , L. Moroni , and S. Camarero‐Espinosa , “Bioprinting Vasculature: Materials, Cells and Emergent Techniques,” Materials 12 (2019): 2701. 31450791 10.3390/ma12172701 PMC6747573

[134] B. V. Fearing , P. A. Hernandez , L. A. Setton , and N. O. Chahine , “Mechanotransduction and Cell Biomechanics of the Intervertebral Disc,” JOR Spine 1 (2018): e1026. 30569032 10.1002/jsp2.1026 PMC6296470

[135] H. Qiao , K. Zhang , and J. Zhao , “Mechanobiology of Intervertebral Disc Degeneration: From Pathological Mechanisms to Therapeutic Approaches,” Mechanobiology in Medicine 3 (2025): 100162. 41399695 10.1016/j.mbm.2025.100162 PMC12702059

[136] H. T. J. Gilbert , N. Hodson , P. Baird , S. M. Richardson , and J. A. Hoyland , “Acidic pH Promotes Intervertebral Disc Degeneration: Acid‐Sensing Ion Channel‐3 as a Potential Therapeutic Target,” Scientific Reports 6 (2016): 37360. 27853274 10.1038/srep37360 PMC5112591

[137] G. W. D. Easson , A. Savadipour , C. Gonzalez , F. Guilak , and S. Y. Tang , “TRPV4 Differentially Controls Inflammatory Cytokine Networks During Static and Dynamic Compression of the Intervertebral Disc,” JOR Spine 6 (2023): e1282. 38156056 10.1002/jsp2.1282 PMC10751971

[138] M. Mengoni , F. Y. Zapata‐Cornelio , V. N. Wijayathunga , and R. K. Wilcox , “Experimental and Computational Comparison of Intervertebral Disc Bulge for Specimen‐Specific Model Evaluation Based on Imaging,” Frontiers in Bioengineering and Biotechnology 9 (2021): 661469. 34124021 10.3389/fbioe.2021.661469 PMC8193738

[139] A. A. Dayem , S. B. Lee , K. M. Lim , et al., “Bioactive Peptides for Boosting Stem Cell Culture Platform: Methods and Applications,” Biomedicine & Pharmacotherapy 160 (2023): 114376. 36764131 10.1016/j.biopha.2023.114376

[140] L. A. Low , C. Mummery , B. R. Berridge , C. P. Austin , and D. A. Tagle , “Organs‐on‐Chips: Into the Next Decade,” Nature Reviews Drug Discovery 20 (2021): 345–361. 32913334 10.1038/s41573-020-0079-3

[141] I. L. M. Isa , B. Günay , K. Joyce , and A. Pandit , “Tissue Engineering: Biomaterials for Disc Repair,” Current Molecular Biology Reports 4 (2018): 161–172.

[142] T. Ma , C. Liu , Q. Zhao , Y. Zhang , and L. Xiao , “Decellularized Nucleus Pulposus Matrix/Chitosan Hybrid Hydrogel Combined With Nucleus Pulposus Stem Cells and GDF5‐Loaded Microspheres for Intervertebral Disc Degeneration Prevention,” Molecular Medicine 30 (2024): 7. 38200442 10.1186/s10020-024-00777-z PMC10782726

[143] M. V. Risbud , Z. R. Schoepflin , F. Mwale , et al., “Defining the Phenotype of Young Healthy Nucleus Pulposus Cells: Recommendations of the Spine Research Interest Group at the 2014 Annual ORS Meeting,” Journal of Orthopaedic Research 33 (2015): 283–293. 25411088 10.1002/jor.22789 PMC4399824

[144] J. Wang , Y. Huang , L. Huang , et al., “Novel Biomarkers of Intervertebral Disc Cells and Evidence of Stem Cells in the Intervertebral Disc,” Osteoarthritis and Cartilage 29 (2021): 389–401. 33338640 10.1016/j.joca.2020.12.005

[145] I. L. Mohd Isa , I. Zulkiflee , R. H. Ogaili , et al., “Three‐Dimensional Hydrogel With Human Wharton Jelly‐Derived Mesenchymal Stem Cells Towards Nucleus Pulposus Niche,” Frontiers in Bioengineering and Biotechnology 11 (2023): 1296531. 38149172 10.3389/fbioe.2023.1296531 PMC10749916

[146] C. Liu , X. Ge , and Y. Li , “Repair of Annulus Fibrosus Defects Using Decellularized Annulus Fibrosus Matrix/Chitosan Hybrid Hydrogels,” Journal of Orthopaedic Surgery and Research 19 (2024): 535. 39223621 10.1186/s13018-024-05017-y PMC11370001

[147] X. Li , R. Huo , L. Li , et al., “Composite Hydrogel Sealants for Annulus Fibrosus Repair,” ACS Biomaterials Science & Engineering 10 (2024): 5094–5107. 38979636 10.1021/acsbiomaterials.4c00548

[148] M. A. H. Tanvir , M. A. Khaleque , J. Lee , et al., “Three‐Dimensional Bioprinting for Intervertebral Disc Regeneration,” Journal of Functional Biomaterials 16 (2025): 105. 40137384 10.3390/jfb16030105 PMC11943008

[149] H. J. Kim , H. R. Lee , H. Kim , and S. H. Do , “Hypoxia Helps Maintain Nucleus Pulposus Homeostasis by Balancing Autophagy and Apoptosis,” Oxidative Medicine and Cellular Longevity 2020 (2020): 5915481. 33029281 10.1155/2020/5915481 PMC7528147

[150] M. V. Risbud , E. Schipani , and I. M. Shapiro , “Hypoxic Regulation of Nucleus Pulposus Cell Survival: From Niche to Notch,” American Journal of Pathology 176 (2010): 1577–1583. 20133815 10.2353/ajpath.2010.090734 PMC2843446

[151] F. Wang , F. Cai , R. Shi , J. N. Wei , and X. T. Wu , “Hypoxia Regulates Sumoylation Pathways in Intervertebral Disc Cells: Implications for Hypoxic Adaptations,” Osteoarthritis and Cartilage 24 (2016): 1113–1124. 26826302 10.1016/j.joca.2016.01.134

[152] M. V. Risbud , A. Guttapalli , D. G. Stokes , et al., “Nucleus Pulposus Cells Express HIF‐1α Under Normoxic Culture Conditions: A Metabolic Adaptation to the Intervertebral Disc Microenvironment,” Journal of Cellular Biochemistry 98 (2006): 152–159. 16408279 10.1002/jcb.20765

[153] G. Feng , L. Li , H. Liu , et al., “Hypoxia Differentially Regulates Human Nucleus Pulposus and Annulus Fibrosus Cell Extracellular Matrix Production in 3D Scaffolds,” Osteoarthritis and Cartilage 21 (2013): 582–588. 23313531 10.1016/j.joca.2013.01.001

[154] S. R. S. Bibby and J. P. G. Urban , “Effect of Nutrient Deprivation on the Viability of Intervertebral Disc Cells,” European Spine Journal 13 (2004): 695–701. 15048560 10.1007/s00586-003-0616-x PMC3454063

[155] T. Grunhagen , G. Wilde , D. M. Soukane , S. A. Shirazi‐Adl , and J. P. G. Urban , “Nutrient Supply and Intervertebral Disc Metabolism,” Journal of Bone and Joint Surgery 88 (2006): 30. 10.2106/JBJS.E.01290 16595440

[156] E. E. McDonnell and C. T. Buckley , “Consolidating and Re‐Evaluating the Human Disc Nutrient Microenvironment,” JOR Spine 5 (2022): e1192. 35386756 10.1002/jsp2.1192 PMC8966889

[157] Z. Meng , J. Tan , X. Ouyang , J. Yin , and Y. Yan , “The Relationship Between Biomechanical Factors and Intervertebral Disc Degeneration: A Review,” American Journal of Translational Research 17 (2025): 3575–3585. 40535657 10.62347/DSJK1156 PMC12170379

[158] R. D. Bowles , H. H. Gebhard , R. Härtl , and L. J. Bonassar , “Tissue‐Engineered Intervertebral Discs Produce New Matrix, Maintain Disc Height, and Restore Biomechanical Function to the Rodent Spine,” Proceedings of the National Academy of Sciences of the United States of America 108 (2011): 13106–13111. 21808048 10.1073/pnas.1107094108 PMC3156186

[159] A. C. Daly , S. E. Critchley , E. M. Rencsok , and D. J. Kelly , “A Comparison of Different Bioinks for 3D Bioprinting of Fibrocartilage and Hyaline Cartilage,” Biofabrication 8 (2016): 045002. 27716628 10.1088/1758-5090/8/4/045002

[160] Z. Liu , H. Wang , Z. Yuan , et al., “High‐Resolution 3D Printing of Angle‐Ply Annulus Fibrosus Scaffolds for Intervertebral Disc Regeneration,” Biofabrication 15 (2023): 015015. 10.1088/1758-5090/aca71f 36541475

[161] A. Ahmed , I. M. Joshi , M. Mansouri , et al., “Engineering Fiber Anisotropy Within Natural Collagen Hydrogels,” American Journal of Physiology – Cell Physiology 320 (2021): C1112–C1124. 33852366 10.1152/ajpcell.00036.2021 PMC8285641

[162] B. Kong , Y. Chen , R. Liu , et al., “Fiber Reinforced GelMA Hydrogel to Induce the Regeneration of Corneal Stroma,” Nature Communications 11 (2020): 1435. 10.1038/s41467-020-14887-9 PMC7080797 32188843

[163] N. Weigel , Y. Li , J. Thiele , and A. Fery , “From Microfluidics to Hierarchical Hydrogel Materials,” Current Opinion in Colloid & Interface Science 64 (2023): 101673.

[164] T. W. Kim , A. G. Kim , K. H. Lee , M. H. Hwang , and H. Choi , “Microfluidic Electroceuticals Platform for Therapeutic Strategies of Intervertebral Disc Degeneration: Effects of Electrical Stimulation on Human Nucleus Pulposus Cells Under Inflammatory Conditions,” International Journal of Molecular Sciences 23 (2022): 10122. 36077518 10.3390/ijms231710122 PMC9456475

[165] N. V. Vo , R. A. Hartman , T. Yurube , L. J. Jacobs , G. A. Sowa , and J. D. Kang , “Expression and Regulation of Metalloproteinases and Their Inhibitors in Intervertebral Disc Aging and Degeneration,” Spine Journal 13 (2013): 331–341. 10.1016/j.spinee.2012.02.027 PMC3637842 23369495

[166] W. J. Wang , X. H. Yu , C. Wang , et al., “MMPs and ADAMTSs in Intervertebral Disc Degeneration,” Clinica Chimica Acta 448 (2015): 238–246. 10.1016/j.cca.2015.06.023 26162271

[167] R. M. Gonçalves , T. Saggese , Z. Yong , et al., “Interleukin‐1β More Than Mechanical Loading Induces a Degenerative Phenotype in Human Annulus Fibrosus Cells, Partially Impaired by Anti‐Proteolytic Activity of Mesenchymal Stem Cell Secretome,” Frontiers in Bioengineering and Biotechnology 9 (2021): 802789. 35155408 10.3389/fbioe.2021.802789 PMC8831733

[168] Z. Yao , L. Nie , Y. Zhao , et al., “Salubrinal Suppresses IL‐17‐Induced Upregulation of MMP‐13 and Extracellular Matrix Degradation Through the NF‐kB Pathway in Human Nucleus Pulposus Cells,” Inflammation 39 (2016): 1997–2007. 27590238 10.1007/s10753-016-0435-y

[169] A. Srivastava , I. L. M. Isa , P. Rooney , and A. Pandit , “Bioengineered Three‐Dimensional Diseased Intervertebral Disc Model Revealed Inflammatory Crosstalk,” Biomaterials 123 (2017): 127–141. 28167391 10.1016/j.biomaterials.2017.01.045

[170] J. Wang , Y. Zhang , Y. Huang , et al., “Application Trends and Strategies of Hydrogel Delivery Systems in Intervertebral Disc Degeneration: A Bibliometric Review,” Materials Today Bio 28 (2024): 101251. 10.1016/j.mtbio.2024.101251 PMC11421353 39318370

[171] R. D. Bowles and L. A. Setton , “Biomaterials for Intervertebral Disc Regeneration and Repair,” Biomaterials 129 (2017): 54–67. 28324865 10.1016/j.biomaterials.2017.03.013 PMC5627607

[172] J. Silva‐Correia , S. I. Correia , J. M. Oliveira , and R. L. Reis , “Tissue Engineering Strategies Applied in the Regeneration of the Human Intervertebral Disk,” Biotechnology Advances 31 (2013): 1514–1531. 23911974 10.1016/j.biotechadv.2013.07.010

[173] B. G. Peng , “Fundamentals of Intervertebral Disc Degeneration and Related Discogenic Pain,” World Journal of Orthopedics 16 (2025): 102119. 39850042 10.5312/wjo.v16.i1.102119 PMC11752479

[174] S. E. Navone , G. Marfia , A. Giannoni , et al., “Inflammatory Mediators and Signalling Pathways Controlling Intervertebral Disc Degeneration,” Histology and Histopathology 32 (2017): 523. 27848245 10.14670/HH-11-846

[175] W. Li , Y. Gong , J. Liu , et al., “Peripheral and Central Pathological Mechanisms of Chronic Low Back Pain: A Narrative Review,” Journal of Pain Research 14 (2021): 1483–1494. 34079363 10.2147/JPR.S306280 PMC8166276

[176] N. F. Zàaba , R. H. Ogaili , F. Ahmad , and I. L. Mohd Isa , “Neuroinflammation and Nociception in Intervertebral Disc Degeneration: A Review of Precision Medicine Perspective,” Spine Journal 25 (2025): 1139–1153. 10.1016/j.spinee.2024.12.033 39814205

[177] S. B. McMahon , “NGF as a Mediator of Inflammatory Pain,” Philosophical Transactions of the Royal Society of London B Biological Sciences 351 (1996): 431–440. 8730782 10.1098/rstb.1996.0039

[178] T. Ohba , H. Haro , T. Ando , et al., “TNF‐α‐Induced NF‐κB Signaling Reverses Age‐Related Declines in VEGF Induction and Angiogenic Activity in Intervertebral Disc Tissues,” Journal of Orthopaedic Research 27 (2009): 229–235. 18683887 10.1002/jor.20727

[179] A. Jabłońska‐Trypuć , M. Matejczyk , and S. Rosochacki , “Matrix Metalloproteinases (MMPs), the Main Extracellular Matrix (ECM) Enzymes in Collagen Degradation, as a Target for Anticancer Drugs,” Journal of Enzyme Inhibition and Medicinal Chemistry 31 (2016): 177–183. 27028474 10.3109/14756366.2016.1161620

[180] D. Purmessur , A. J. Freemont , and J. A. Hoyland , “Expression and Regulation of Neurotrophins in the Nondegenerate and Degenerate Human Intervertebral Disc,” Arthritis Research & Therapy 10 (2008): R99. 18727839 10.1186/ar2487 PMC2575613

[181] A. L. A. Binch , A. A. Cole , L. M. Breakwell , et al., “Expression and Regulation of Neurotrophic and Angiogenic Factors During Human Intervertebral Disc Degeneration,” Arthritis Research & Therapy 16 (2014): 416. 25209447 10.1186/s13075-014-0416-1 PMC4177417

[182] H. Liang , R. Luo , G. Li , W. Zhang , Y. Song , and C. Yang , “The Proteolysis of ECM in Intervertebral Disc Degeneration,” International Journal of Molecular Sciences 23 (2022): 1715. 35163637 10.3390/ijms23031715 PMC8835917

[183] H. J. Moon , J. H. Kim , H. S. Lee , et al., “Annulus Fibrosus Cells Interact With Neuron‐Like Cells to Modulate Production of Growth Factors and Cytokines in Symptomatic Disc Degeneration,” Spine 37 (2012): 2–9. 21386768 10.1097/BRS.0b013e31820cd2d8

[184] P. Sahbaie , X. Shi , T. Z. Guo , et al., “Role of Substance P Signaling in Enhanced Nociceptive Sensitization and Local Cytokine Production After Incision,” Pain 145 (2009): 341–349. 19660865 10.1016/j.pain.2009.06.037 PMC2746201

[185] Z. Li , X. Li , J. Liu , et al., “Molecular Mechanisms of Chronic Pain and Therapeutic Interventions,” MedComm 6 (2025): e70325. 40787071 10.1002/mco2.70325 PMC12331885

[186] S. Ansaryan , Y. C. Liu , X. Li , et al., “High‐Throughput Spatiotemporal Monitoring of Single‐Cell Secretions via Plasmonic Microwell Arrays,” Nature Biomedical Engineering 7 (2023): 943–958. 10.1038/s41551-023-01017-1 PMC10365996 37012313

[187] P. H. Chou , S. T. Wang , M. H. Yen , C. L. Liu , M. C. Chang , and O. K. S. Lee , “Fluid‐Induced, Shear Stress‐Regulated Extracellular Matrix and Matrix Metalloproteinase Genes Expression on Human Annulus Fibrosus Cells,” Stem Cell Research & Therapy 7 (2016): 34. 26921206 10.1186/s13287-016-0292-5 PMC4769486

[188] J. García‐Cosamalón , M. E. Del Valle , M. G. Calavia , et al., “Intervertebral Disc, Sensory Nerves and Neurotrophins: Who Is Who in Discogenic Pain?,” Journal of Anatomy 217 (2010): 1–15. 20456524 10.1111/j.1469-7580.2010.01227.x PMC2913007

[189] K. Sun , J. Jiang , Y. Wang , et al., “The Role of Nerve Fibers and Their Neurotransmitters in Regulating Intervertebral Disc Degeneration,” Ageing Research Reviews 81 (2022): 101733. 36113765 10.1016/j.arr.2022.101733

[190] J. M. Lee , J. Y. Song , M. Baek , et al., “Interleukin‐1β Induces Angiogenesis and Innervation in Human Intervertebral Disc Degeneration,” Journal of Orthopaedic Research 29 (2011): 265–269. 20690185 10.1002/jor.21210

[191] K. Sun , J. Zhu , C. Yan , et al., “CGRP Regulates Nucleus Pulposus Cell Apoptosis and Inflammation via the MAPK/NF‐κB Signaling Pathways During Intervertebral Disc Degeneration,” Oxidative Medicine and Cellular Longevity 2021 (2021): 2958584. 34987701 10.1155/2021/2958584 PMC8720589

[192] J. D. Koerner , D. Z. Markova , G. D. Schroeder , et al., “The Effect of Substance P on an Intervertebral Disc Rat Organ Culture Model,” Spine 41 (2016): 1851–1859. 27163370 10.1097/BRS.0000000000001676

[193] A. J. Freemont , T. E. Peacock , P. Goupille , J. A. Hoyland , J. O’Brien , and M. I. Jayson , “Nerve Ingrowth Into Diseased Intervertebral Disc in Chronic Back Pain,” Lancet 350 (1997): 178–181. 9250186 10.1016/s0140-6736(97)02135-1

[194] A. L. A. Binch , A. A. Cole , L. M. Breakwell , et al., “Nerves Are More Abundant Than Blood Vessels in the Degenerate Human Intervertebral Disc,” Arthritis Research & Therapy 17 (2015): 370. 26695177 10.1186/s13075-015-0889-6 PMC4704545

[195] M. Campisi , Y. Shin , T. Osaki , C. Hajal , V. Chiono , and R. D. Kamm , “3D Self‐Organized Microvascular Model of the Human Blood‐Brain Barrier With Endothelial Cells, Pericytes and Astrocytes,” Biomaterials 180 (2018): 117–129. 30032046 10.1016/j.biomaterials.2018.07.014 PMC6201194

[196] A. Cuesta , E. Viña , R. Cabo , et al., “Acid‐Sensing Ion Channel 2 (ASIC 2) and Trkb Interrelationships Within the Intervertebral Disc,” International Journal of Clinical and Experimental Pathology 8 (2015): 10305–10314. 26617738 PMC4637553

[197] J. D. Stover , B. Lawrence , and R. D. Bowles , “Degenerative IVD Conditioned Media and Acidic pH Sensitize Sensory Neurons to Cyclic Tensile Strain,” Journal of Orthopaedic Research 39 (2021): 1192–1203. 32255531 10.1002/jor.24682 PMC9265139

[198] M. K. M. Kim , M. Lawrence , D. Quinonez , C. Brooks , R. Ramachandran , and C. A. Séguin , “Transient Receptor Potential Vanilloid 4 Regulates Extracellular Matrix Composition and Mediates Load‐Induced Intervertebral Disc Degeneration in a Mouse Model,” Osteoarthritis and Cartilage 32 (2024): 881–894. 38604493 10.1016/j.joca.2024.04.001

[199] T. Matsuo , Y. Takeoka , T. Yurube , et al., “Transient Receptor Potential Vanilloid 4 Knockdown Decreases Extracellular Matrix Synthesis via Autophagy Suppression in the Rat Intervertebral Disc,” JOR Spine 8 (2025): e70046. 39963549 10.1002/jsp2.70046 PMC11832302

[200] H. Brisby , “Pathology and Possible Mechanisms of Nervous System Response to Disc Degeneration,” Journal of Bone and Joint Surgery 88 (2006): 68–71. 10.2106/JBJS.E.01282 16595447

[201] D. P. Jacobsen , A. Moen , F. Haugen , and J. Gjerstad , “Hyperexcitability in Spinal WDR Neurons Following Experimental Disc Herniation Is Associated With Upregulation of Fractalkine and Its Receptor in Nucleus Pulposus and the Dorsal Root Ganglion,” International Journal of Inflammation 2016 (2016): 6519408. 28116212 10.1155/2016/6519408 PMC5220471

[202] C. J. Woolf , “Central Sensitization: Implications for the Diagnosis and Treatment of Pain,” supplement, Pain 152 (2011): S2–S15. 20961685 10.1016/j.pain.2010.09.030 PMC3268359

[203] F. Zipp , S. Bittner , and D. P. Schafer , “Cytokines as Emerging Regulators of Central Nervous System Synapses,” Immunity 56 (2023): 914–925. 37163992 10.1016/j.immuni.2023.04.011 PMC10233069

[204] C. L. Le Maitre , A. J. Freemont , and J. A. Hoyland , “The Role of Interleukin‐1 in the Pathogenesis of Human Intervertebral Disc Degeneration,” Arthritis Research & Therapy 7 (2005): R732–R745. 15987475 10.1186/ar1732 PMC1175026

[205] S. N. Raja , D. B. Carr , M. Cohen , et al., “The Revised IASP Definition of Pain: Concepts, Challenges, and Compromises,” Pain 161 (2020): 1976–1982. 32694387 10.1097/j.pain.0000000000001939 PMC7680716

[206] Y. Aoki , A. Nakajima , S. Ohtori , et al., “Increase of Nerve Growth Factor Levels in the Human Herniated Intervertebral Disc: Can Annular Rupture Trigger Discogenic Back Pain?,” Arthritis Research & Therapy 16 (2014): R159. 25069717 10.1186/ar4674 PMC4261264

[207] H. J. Moon , T. Yurube , T. P. Lozito , et al., “Effects of Secreted Factors in Culture Medium of Annulus Fibrosus Cells on Microvascular Endothelial Cells: Elucidating the Possible Pathomechanisms of Matrix Degradation and Nerve In‐Growth in Disc Degeneration,” Osteoarthritis and Cartilage 22 (2014): 344–354. 24361793 10.1016/j.joca.2013.12.008 PMC3952937

[208] S. Roberts , E. H. Evans , D. Kletsas , D. C. Jaffray , and S. M. Eisenstein , “Senescence in Human Intervertebral Discs,” supplement, European Spine Journal 15 (2006): 312–316. 10.1007/s00586-006-0126-8 PMC2335374 16773379

[209] H. E. Gruber , J. A. Ingram , H. J. Norton , and E. N. Hanley , “Senescence in Cells of the Aging and Degenerating Intervertebral Disc: Immunolocalization of Senescence‐Associated β‐Galactosidase in Human and Sand Rat Discs,” Spine 32 (2007): 321–327. 17268263 10.1097/01.brs.0000253960.57051.de

[210] G. Vadalà , F. Russo , L. Ambrosio , M. Loppini , and V. Denaro , “Stem Cells Sources for Intervertebral Disc Regeneration,” World Journal of Stem Cells 8 (2016): 185–201. 27247704 10.4252/wjsc.v8.i5.185 PMC4877563

[211] M. Bekbolatova , J. Mayer , C. W. Ong , and M. Toma , “Transformative Potential of AI in Healthcare: Definitions, Applications, and Navigating the Ethical Landscape and Public Perspectives,” Healthcare 12 (2024): 125. 38255014 10.3390/healthcare12020125 PMC10815906

[212] X. Hu , Z. Wang , H. Zhang , et al., “Single‐Cell Sequencing: New Insights for Intervertebral Disc Degeneration,” Biomedicine & Pharmacotherapy 165 (2023): 115224. 37516017 10.1016/j.biopha.2023.115224

[213] N. F. Za`aba , R. Razak , I. Zulkiflee , et al., “Human Cadaveric Spine Dissection: A Protocol for Harvesting Lumbar Intervertebral Discs From Body Donors,” Annals of Anatomy‐Anatomischer Anzeiger 260 (2025): 152680. 10.1016/j.aanat.2025.152680 40419030

[214] A. L. Paguirigan and D. J. Beebe , “Microfluidics Meet Cell Biology: Bridging the Gap by Validation and Application of Microscale Techniques for Cell Biological Assays,” BioEssays 30 (2008): 811. 18693260 10.1002/bies.20804 PMC2814162

